# Regulatory NLRs Control the RLR-Mediated Type I Interferon and Inflammatory Responses in Human Dendritic Cells

**DOI:** 10.3389/fimmu.2018.02314

**Published:** 2018-10-05

**Authors:** Tünde Fekete, Dora Bencze, Attila Szabo, Eszter Csoma, Tamas Biro, Attila Bacsi, Kitti Pazmandi

**Affiliations:** ^1^Department of Immunology, Faculty of Medicine, University of Debrecen, Debrecen, Hungary; ^2^Department of Medical Microbiology, Faculty of Medicine, University of Debrecen, Debrecen, Hungary

**Keywords:** RLR, NLR, regulate, dendritic cell, interferon, antiviral

## Abstract

Unique members of the nucleotide-binding domain leucine-rich repeat (NLR) family have been found to regulate intracellular signaling pathways initiated by other families of pattern recognition receptors (PRR) such as Toll-like receptors (TLRs) and retinoic-acid inducible gene I (RIG-I)-like receptors (RLRs). Plasmacytoid dendritic cells (pDCs), the most powerful type I interferon (IFN) producing cells, preferentially employ endosomal TLRs to elicit antiviral IFN responses. By contrast, conventional DCs (cDCs) predominantly use cytosolic RLRs, which are constitutively expressed in them, to sense foreign nucleic acids. Previously we have reported that, though RIG-I is absent from resting pDCs, it is inducible upon TLR stimulation. In the recent study we investigated the regulatory ability of NLRs, namely NLRC5 and NLRX1 directly associated with the RLR-mediated signaling pathway in DC subtypes showing different RLR expression, particularly in pDCs, and monocyte-derived DCs (moDCs). Here we demonstrate that similarly to RLRs, NLRC5 is also inducible upon TLR9 stimulation, whereas NLRX1 is constitutively expressed in pDCs. Inhibition of NLRC5 and NLRX1 expression in pDCs augmented the RLR-stimulated expression of type I IFNs but did not affect the production of the pro-inflammatory cytokines TNF, IL-6, and the chemokine IL-8. Further we show that immature moDCs constantly express RLRs, NLRX1 and NLRC5 that are gradually upregulated during their differentiation. Similarly to pDCs, NLRX1 suppression increased the RLR-induced production of type I IFNs in moDCs. Interestingly, RLR stimulation of NLRX1-silenced moDCs leads to a significant increase in pro-inflammatory cytokine production and IκBα degradation, suggesting increased NF-κB activity. On the contrary, NLRC5 does not seem to have any effect on the RLR-mediated cytokine responses in moDCs. In summary, our results indicate that NLRX1 negatively regulates the RLR-mediated type I IFN production both in pDCs and moDCs. Further we show that NLRX1 inhibits pro-inflammatory cytokine secretion in moDCs but not in pDCs following RLR stimulation. Interestingly, NLRC5 suppresses the RLR-induced type I IFN secretion in pDCs but does not appear to have any regulatory function on the RLR pathway in moDCs. Collectively, our work demonstrates that RLR-mediated innate immune responses are primarily regulated by NLRX1 and partly controlled by NLRC5 in human DCs.

## Introduction

DCs, acting as sentinels of the immune system, recognize various molecular motifs within pathogens through their PRRs and rapidly produce inflammatory cytokines and/or antiviral molecules to initiate innate immune responses (1). In order to fulfill this task, DCs are equipped with an arsenal of germ-line encoded PRRs including TLRs, RLRs and NLRs (2). Recent evidence indicates that these PRRs might collaborate synergistically to counteract the infectious agents or antagonistically to attenuate overzealous inflammation (3–5). In this report our primary goal was to reveal possible interactions between RLRs and NLRs in different human DC subtypes.

RLRs function as cytoplasmic sensors of viral RNA and trigger type I IFN production and antiviral gene expression to control viral infection (6–8). To date, three RLR members have been identified: RIG-I, Melanoma differentiation-associated gene-5 (MDA5), and their regulatory molecule, Laboratory of genetics and physiology 2 (LGP2), which are broadly expressed in most tissues (6, 9). RIG-I, the prototypical member of the RLR family, preferentially recognizes short RNA sequences marked with 5′ triphosphorylated (5′ppp) ends (10). In contrast to RIG-I, MDA5 predominantly recognizes long dsRNAs (11). Although many natural ligands including incoming viral nucleocapsids (12), virus genomes (13), virus replication intermediates (14), or viral transcripts (15, 16) are recognized by RIG-I (10), genomic RNA generated by viral replication seems to constitute the major trigger (17). These findings suggest that RIG-I stimulation requires the presence of actively replicating viruses, in contrast to endosomal TLRs, which are mainly activated by internalized viruses, phagocytosed infected materials or apoptotic cell debris (18, 19).

Upon activation, RLRs translocate to mitochondria where they interact with the mitochondrial antiviral signaling adaptor (MAVS). RLR-MAVS interaction leads to the recruitment of downstream signaling factors, such as inhibitor of kappa kinases (IKK) and Tank-binding kinase 1 (TBK1) which activate nuclear factor-κB (NF-κB) and interferon regulatory factor (IRF) 3 that are crucial for the induction of type I IFNs, inflammatory cytokines and chemokines, respectively (6, 10). RIG-I and MDA5 have been found to be differently expressed by distinct DC subtypes; they are constitutively expressed in cDCs and macrophages, whereas absent or maintained at low levels in resting pDCs (20–22). It has been suggested that pDCs mainly rely on endosomal TLRs for recognition of viruses whereas other cells such as cDCs preferentially use cytosolic RLRs to recognize replicating viral RNA intermediates (23, 24). Intriguingly it has been found that in the absence of IFN positive feedback mouse pDCs can mount an antiviral response through a synergistic TLR- and RLR-dependent recognition and type I IFN production (25). In line with this, we recently reported that pDCs express RIG-I at very low level under steady-state conditions; however its expression can be greatly upregulated by endosomal TLR stimulation in a type I IFN-independent manner (26). We have also proposed a model where endosomal TLRs mediate the early phase of type I IFN production in pDCs while RIG-I participates in the late phase of IFN responses (27).

NLRs constitute a large family of intracellular PRRs, that can be divided into four functional categories: signal transduction, inflammasome assembly, transcriptional activation and autophagy (28). Recently certain members of the NLR family, known as regulatory NLRs, have been found to modulate diverse signaling pathways including the NF-κB, mitogen-activated protein kinase (MAPK) and type I IFN responses (4, 29). The role of regulatory NLRs is widely studied in animal models, but little is known about their regulatory functions in human cells, especially in DCs, which are essential for both innate and adaptive antiviral responses. However, to maintain immune balance NF-κB and type I IFN signaling must be tightly regulated in these cells (30). So far two regulatory NLRs, namely NLRC5 and NRLX1, have been assigned to the RIG-I-mediated signaling pathway, though conflicting results have been reported with regard to their role in the regulation of antiviral innate immune responses (31).

In this study our goal was to explore the expression profile of these regulatory NLRs and to reveal their contribution to the RLR-mediated cytokine responses using a human pDC cell line and human moDCs.

## Materials and methods

### Cell lines and culture conditions

The human pDC cell line (GEN2.2) was used in our experiments which was generated by Joël Plumas and Laurence Chaperot (32), researchers of the French National Blood Service (Etablissement Français du Sang, EFS) and was deposited with the CNCM (French National Collection of Microorganism Cultures) under the number CNCMI-2938. The GEN2.2 cell line was grown on a layer of mitomycin C (Sigma-Aldrich, St. Louis, MO, USA, Cat. No. M4287)-treated murine MS5 feeder cells (ACC 441, Leibniz Institute DSMZ-German Collection of Microorganisms and Cell Cultures, Braunschweig, Germany) in RPMI 1640 medium (Sigma-Aldrich, Cat. No. R8758) supplemented with 10% heat-inactivated FBS (Life Technologies Corporation, Carlsbad, CA, USA, Cat. No. 10270-106), 100 U/ml penicillin, 100 μg/ml streptomycin (both from Biosera, Nuaille, France, Cat. No. XC-A4122/100) and 5% non-essential amino acids (Life Technologies Corporation, Cat. No. 11140050). For experiments, the GEN2.2 cells were removed from the feeder layer, subjected to small interfering RNA (siRNA) transfection then seeded on 24-well plates at a concentration of 5 × 10^5^ cells/500 μl in RPMI 1640 medium (Sigma-Aldrich, Cat. No. R8758). Cells were grown and incubated at 37°C in 5% CO_2_ humidified atmosphere.

For virus propagation African green monkey kidney epithelial Vero cell line (ATCC-CCL-81; The American Type Culture Collection [ATCC], Manassas, VA, USA) was used. Vero cells were grown in Dulbecco's modified Eagle's minimal essential medium (DMEM; Sigma-Aldrich, Cat. No. D6546) supplemented with 10% heat-inactivated FBS (Life Technologies Corporation, Cat. No. 10270-106), 100 U/ml penicillin, 100 μg/ml streptomycin (both from Biosera, Cat. No. XC-A4122/100) at 37°C in a 5% CO_2_ humidified atmosphere.

### Primary cell isolation and culture

Human heparinized leukocyte-enriched buffy coats were obtained from healthy blood donors drawn at the Regional Blood Center of Hungarian National Blood Transfusion Service (Debrecen, Hungary) in accordance with the written approval of the Director of the National Blood Transfusion Service and the Regional and Institutional Ethics Committee of the University of Debrecen, Faculty of Medicine (Debrecen, Hungary).

Peripheral blood mononuclear cells (PBMC) were separated from buffy coats by Ficoll-Paque Plus (GE Healthcare, Little Chalfont, Buckinghamshire, UK, Cat. No. 17-1440-03) gradient centrifugation.

Monocytes were purified from PBMCs by positive selection using magnetic cell separation with anti-CD14-conjugated microbeads (Miltenyi Biotech, Bergish Gladbach, Germany, Cat. No. 130-050-201). Freshly isolated cells were subjected to siRNA transfection then seeded in 24-well cell culture plates at a density of 10^6^ cells/ml in RPMI 1640 medium (Sigma-Aldrich, Cat. No. R8758) supplemented with 10% heat-inactivated FBS (Life Technologies Corporation, Cat. No. 10270-106), 2 mM L-glutamine (Biosera, Cat. No. XC-T1755/100), 100 U/ml penicillin, 100 μg/ml streptomycin (both from Biosera, Cat. No. XC-A4122/100), 80 ng/ml GM-CSF (Gentaur Molecular Products, London, UK, Cat. No. 04-RHUGM-CSF-300 MCG) and 50 ng/ml IL-4 (PeproTech, Brussels, Belgium, Cat. No. 200-04) for 5 days.

Human pDCs were isolated from PBMCs by positive selection using the human CD304 (BDCA-4/Neuropilin-1) MicroBead Kit (Miltenyi Biotech, Cat. No. 130-090-532) then cultured in 48-well cell culture plates at a density of 5 × 10^5^ cells/500 μl in RPMI 1640 medium (Sigma-Aldrich, Cat. No. R8758) supplemented with 10% heat-inactivated FBS (Life Technologies Corporation, Cat. No. 10270-106), 2 mM L-glutamine (Biosera, Cat. No. XC-T1755/100), 100 U/ml penicillin, 100 μg/ml streptomycin (both from Biosera, Cat. No. XC-A4122/100), and 50 ng/ml recombinant human IL-3 (Peprotech EC, Cat. No. AF-200-03).

Cells were incubated at 37°C in 5% CO_2_ humidified atmosphere.

### Virus propagation and determination of viral titer

Vesicular Stomatitis Virus (VSV; Indiana serotype), kindly provided by Dr. Eszter Csoma (Department of Medical Microbiology, University of Debrecen, Debrecen, Hungary), was propagated in Vero cell line for 36 h at 37°C. The supernatants of infected cell cultures were harvested and the intact cells or cell debris were removed by centrifugation at 1000 × g for 10 min at 4°C. After filtration of the supernatants using 0.45 μm sterile syringe filter (Rephile, Bioscience Ltd., Shanghai, China, Cat. No. RJF1345NH), the viral supernatants were transferred to Amicon Ultra-15 100K centrifugal filter units (Millipore, Danvers, MA, USA, Cat. No. UFC910024) and spinned at 4000 × g for 20 min at 4°C. The concentrated virus was stored in aliquots at −80°C and used as the infecting stock of the virus.

Plaque assay was performed to determine the viral titer of the virus stocks. Vero cells were seeded in 12-well tissue culture plates and the confluent monolayer of the cells were inoculated with 10-fold serial dilutions of the VSV stock for 1 h at 37°C then overlaid with 0.3% agarose (Sigma-Aldrich, Cat. No. A9539) in DMEM (Sigma-Aldrich, Cat. No. D6546) supplemented with 2% heat-inactivated FBS (Life Technologies Corporation, Cat. No. 10270-106), 100 U/ml penicillin and 100 μg/ml streptomycin (both from Biosera, Cat. No. XC-A4122/100). After 2 days of culturing at 37°C the cells were fixed by 4% formaldehyde (Sigma-Aldrich, Cat. No. F8775) for 1 h at room temperature then the agarose layer was removed. To visualize the plaque formation 0.2% crystal violet (Sigma-Aldrich, Cat. No. 46364) solution was added to the wells for 5 min. The plates were washed and dried, and the number of plaque-forming units per milliliter was calculated.

For the VSV infection of human DCs, virus stocks were diluted to the indicated multiplicity of infection (MOI) and were added to cells for 18 or 24 h.

### SiRNA-mediated gene silencing

GEN2.2. cells or freshly isolated monocytes were left untreated (ctrl), transfected with no siRNA (mock), NLRX1- (Assay ID: s36063, Life Technologies, Cat. No. 4392420) and NLRC5 (Assay ID: s38591, Life Technologies, Cat. No. 4392420)-specific Silencer Select Validated siRNAs and Silencer Select Negative Control siRNA (scr; Life Technologies, Cat. No. 4390844) in Opti-MEM medium (Life Technologies, Cat. No. 11058021) in 4-mm cuvettes (Bio-Rad Laboratories GmbH, Munich, Germany, Cat. No. 1652088) using GenePulser Xcell instrument (Bio-Rad). Following transfection the cells were seeded as described previously.

### Cell stimulation

To induce RIG-I expression GEN2.2 cells were incubated with 0.25 μM CpG-A (ODN 2216; Hycult Biotech, Uden, The Netherlands, Cat. No. HC4037) for 16 h. Thereafter the cells were washed, re-seeded in 24-well plates in fresh, complete RPMI 1640 medium and stimulated with 5′ppp-dsRNA (InvivoGen, San Diego, CA, USA, Cat. No. tlrl-3prna-100), a specific agonist of RIG-I or polyI:C-HMW (HMW: high molecular weight; InvivoGen, Cat. No. tlrl-pic), a RIG-I/MDA5 agonist, both complexed with the transfection reagent LyoVec^TM^ (InvivoGen, Cat. No. lyec-1), according to the manufacturer's recommendations. To induce RIG-I in primary pDCs, cells were treated with 2.5 μM CpG-A for 16 h prior to western blot analysis. In separate experiments, GEN2.2 cells were exposed to 1 or 2 μM of CpG-B (ODN 2006; Hycult Biotech, Cat. No. HC4039), and 1 or 2 μg/ml of Imiquimod (InvivoGen, Cat. No. tlrl-imqs) or PAM3CSK4 (InvivoGen, Cat. No. tlrl-pms) for 24 h. For moDCs, half of the medium was removed, replaced by fresh medium then stimulation with 5′ppp-dsRNA/LyoVec^TM^ or polyI:C-HMW/LyoVec^TM^ complex was performed as described for GEN2.2 cells. For live virus infection untreated and CpG-A pre-treated GEN2.2 cells and moDCs were infected with VSV at a MOI of 1 and 10, respectively for 18 or 24 h.

### Flow cytometry

Phenotypical analysis of moDCs was performed by flow cytometry using anti-CD209-FITC (Cat. No. 33013, Clone: 9E9A8), anti-CD40-FITC (Cat. No. 334306, Clone: 5C3), anti-CD80-FITC (Cat. No. 305206, Clone: 2D10), anti-HLA-DR-FITC (Cat. No. 327006, Clone: LN3), anti-CD14-PE (Cat. No. 367104, Clone: 63D3), anti-CD11c-PE (Cat. No. 301606, Clone: 3.9), anti-CD83-PE-Cy5 (Cat. No. 305310, Clone: HB15e), anti-CD1a-APC (Cat. No. 300110, Clone: HI149), anti-CD1c-APC (Cat. No. 331524, Clone: L161) and their isotype-matched control antibodies (all from BioLegend, San Diego, CA, USA). The viability of electroporated moDCs was also assessed by flow cytometry using 7-aminoactinomycin-D (7-AAD; 10 μg/ml; Sigma-Aldrich, Cat. No. A9400) staining.

Fluorescence intensities were measured with FACSCalibur cytometer (BD Biosciences, Franklin Lakes, NJ, USA) and data were analyzed with FlowJo software (Tree Star, Ashland, OR, USA). Throughout data acquisition, 10,000 events were acquired from each sample containing 100,000 stained cells. Cells were gated on forward vs. side scatter to exclude cell debris. Isotype controls were used to set gates for positive staining of CD14, CD209, CD1a, CD1c, and CD11c. For 7-AAD staining the unstained cells were used as negative control and the percentages of 7-AAD negative live cells were determined by excluding the 7-AAD positive necrotic cell population. Delta median fluorescence intensity values (MFI) of CD40, CD80, CD83, and HLA-DR were obtained by subtracting the MFI values of the isotype control samples from the MFI of the positively stained samples.

### Quantitative real time PCR

Total RNA was isolated from 5 × 10^5^ cells using Tri reagent (Molecular Research Center, Inc., Cincinnati, OH, USA, Cat. No. TR118). Total RNA was treated with DNase I (Thermo Fisher Scientific, Waltham, MA, USA, Cat. No. AM2222) to exclude amplification of genomic DNA then reverse transcribed into cDNA using the High Capacity cDNA RT Kit of Applied Biosystems (Carlsbad, CA, USA, Cat. No. 4368813). Gene expression assays were purchased from Thermo Fisher Scientific for *NLRC5* (Assay ID: Hs01072148_m1, Cat. No. 4331182), *NLRX1* (Assay ID: Hs00226360_m1, Cat. No. 4331182), *IFNB* (Assay ID: Hs01077958_s1, Cat. No. 4331182) and Integrated DNA Technologies (Coralville, IA, USA) for *IFNA1* (Assay ID: Hs.PT.49a.3184790.g) and *PPIA* (cyclophilin A; Assay ID: Hs.PT.58v.38887593.g). Quantitative PCR was performed using the ABI StepOne Real-Time PCR System (Applied Biosystems) and cycle threshold values were determined using the StepOne v2.1 Software (Applied Biosystems). The relative amount of mRNA (2^−ΔCT^) was obtained by normalizing to the *PPIA* housekeeping gene in each experiment.

### Western blotting

Protein extraction was performed by lysing the cells in Laemmli sample buffer and separated by SDS-PAGE using 7.5% polyacrylamide gels and electrotransferred to nitrocellulose membranes (Bio-Rad, Cat. No. 162-0115). Non-specific binding sites were blocked with 5% non-fat dry milk diluted in TBS Tween buffer (50 mM Tris, 0.5 M NaCl, 0.05% Tween-20, pH 7.4). The following antibodies were used for protein detection: anti-RIG-I (Cell Signaling, Danvers, MA, USA, Cat. No. 3743), anti-MDA5 (Cell Signaling, Cat. No. 5321), anti-TBK1 (Cell Signaling, Cat. No. 3504), anti-MAVS (Cell Signaling, Cat. No. 3993), anti-NLRC5 (clone 3H8, Millipore, Cat. No. MABF260), anti-NLRX1 (Proteintech Group, Manchester, UK, Cat. No. 17215-1-AP), anti-IκBα (Cell Signaling, Cat. No. 4812), and anti-β-actin (Santa Cruz Biotechnology, Cat. No. sc-47778). The bound antibodies were labeled with anti-mouse (Bio-Rad, Cat. No. 1721011), anti-rat (Bio-Rad, Cat. No. 5204-2504) or anti-rabbit (GE Healthcare, Cat. No. NA934) horseradish peroxidase-conjugated secondary antibodies and were visualized by the ECL system using SuperSignal West Pico or Femto chemiluminescent substrates (Thermo Scientific, Rockford, IL, USA, Cat. No. 34580 and 34095) and X-ray film exposure. Densitometric analysis of immunoreactive bands was performed using Image Studio Lite Software version 5.2 (LI-COR Biosciences, Lincoln, Nebraska USA).

### ELISA

Cell culture supernatants were collected at the indicated time points and the TNF (Cat. No. 555212), IL-6 (Cat. No. 555220) and IL-8 (Cat. No. 555244) levels were determined by the BD OptEIA human ELISA kits (all from BD Biosciences, San Diego, CA, USA). IFN-α and IFN-β levels were measured by the VeriKine^TM^ Human Interferon Alpha (Cat. No. RD-41100-1) and Interferon Beta (Cat. No. RD-41410-1) ELISA kits, respectively (PBL Interferon Sources, Piscataway, NJ, USA). Assays were performed according to the manufacturer's instructions. Absorbance measurements were carried out by a Synergy HT microplate reader (Bio-Tek Instruments, Winooski, VT, USA) at 450 nm.

### Statistical analysis

Data are expressed as the Mean ± SD and analyzed by Student's unpaired *t*-test or ANOVA, followed by Bonferroni *post hoc* analysis for least-significant differences. Data analysis was performed with GraphPad Prism v.6. Software (GraphPad Software Inc., La Jolla, CA, USA). All experiments were repeated at least three times. Differences were considered to be statistically significant at *P* < 0.05.

## Results

### The expression of NLRC5 and RLRs but not of NLRX1 is upregulated by CpG-A treatment in the human GEN2.2 pDC cell line and in primary human pDCs

**Figure 1:**
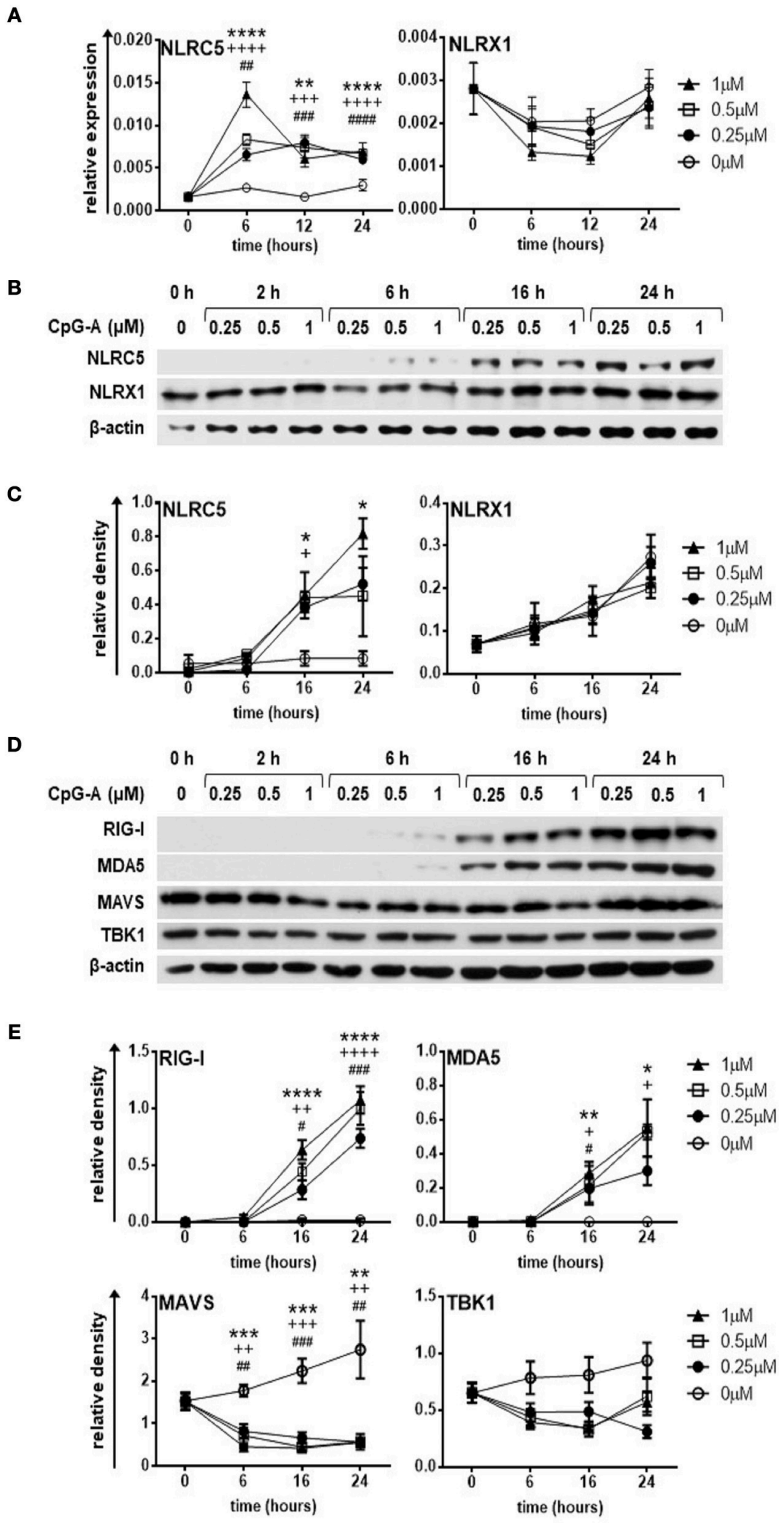
The expression of NLRC5, RIG-I and MDA5 but not that of NLRX1 is upregulated by CpG-A treatment in the human GEN2.2 pDC cell line. **(A–E)** GEN2.2 cells were treated with increasing concentration of CpG-A (0.25–1 μM) in a time dependent manner. The expression of NLRC5 and NLRX1 was measured at the mRNA level by Q-PCR **(A)** and at the protein level by western blotting **(B,C)**. The changes in protein levels of RIG-I, MDA5, MAVS, and TBK1 were also analyzed after CpG-A treatments by western blotting **(D,E)**. Representative blots are shown in **(B,D)**. Data are shown as mean ± SD from 4 to 6 independent experiments in panels **(A,C,E)**. Data were analyzed using one-way ANOVA followed by Bonferroni's *post-hoc* test. ^*^p < 0.05, ^**^p < 0.01, ^***^p < 0.001, ^****^p < 0.0001 1 μM CpG-A vs control; ^+^*p* < 0.05, ^++^*p* < 0.01, ^+++^*p* < 0.001, ^++++^*p* < 0.0001 0.5 μM CpG-A vs control; ^#^*p* < 0.05, ^##^*p* < 0.01, ^###^*p* < 0.001, ^####^*p* < 0.0001 0.25 μM CpG-A vs control.

Human pDCs constitute a very rare cell population in peripheral blood. Therefore, we performed most of our experiments with the human pDC cell line called GEN2.2, which displays similar phenotypic and functional properties to human primary pDCs (33, 34). First, we sought to investigate the expression of NLRC5 and NLRX1 in resting and CpG-A stimulated pDCs. To this end, GEN2.2 cells were stimulated with increasing concentrations of the TLR9 ligand, CpG-A (0.25, 0.5, 1 μM) in a time-dependent manner. Our results show that NLRC5 is not expressed in resting cells but appears both at the mRNA (Figure 1A) and protein level (Figures 1B,C) upon exposure to all applied doses of CpG-A showing a significant upregulation as early as 16 h. In contrast, NLRX1 is constitutively expressed and is not affected by CpG-A stimulation (Figures 1A-C).

In this set of experiments, we also tested the expression of RLRs and downstream signaling molecules (Figures 1D,E). Both RIG-I and MDA5 show a strong upregulation at 16 h upon treatment with even the smallest dose of CpG-A, which correlates with our previous data (27). Interestingly, synthetic ssRNAs and dsRNAs have been found to downregulate MAVS expression (35). In line with this data, we also observed decreased protein levels of MAVS upon CpG-A treatment of GEN2.2 cells, the mechanism of which might be essential to avoid overwhelming host inflammatory responses. On the other hand, TBK1, a key regulator of IFN signaling, was not affected by CpG-A stimulation. As we have previously described 0.25 μM of CpG-A is able to upregulate RIG-I expression in GEN2.2 cells in a way that it does not induce IFN production, and therefore does not result in cell exhaustion (27). Since RIG-I, MDA5 and NLRC5 are greatly upregulated upon treatment with 0.25 μM of CpG-A at 16 h, we decided to use these culture conditions in our further experiments with the GEN2.2 cell line.

**Figure 2:**
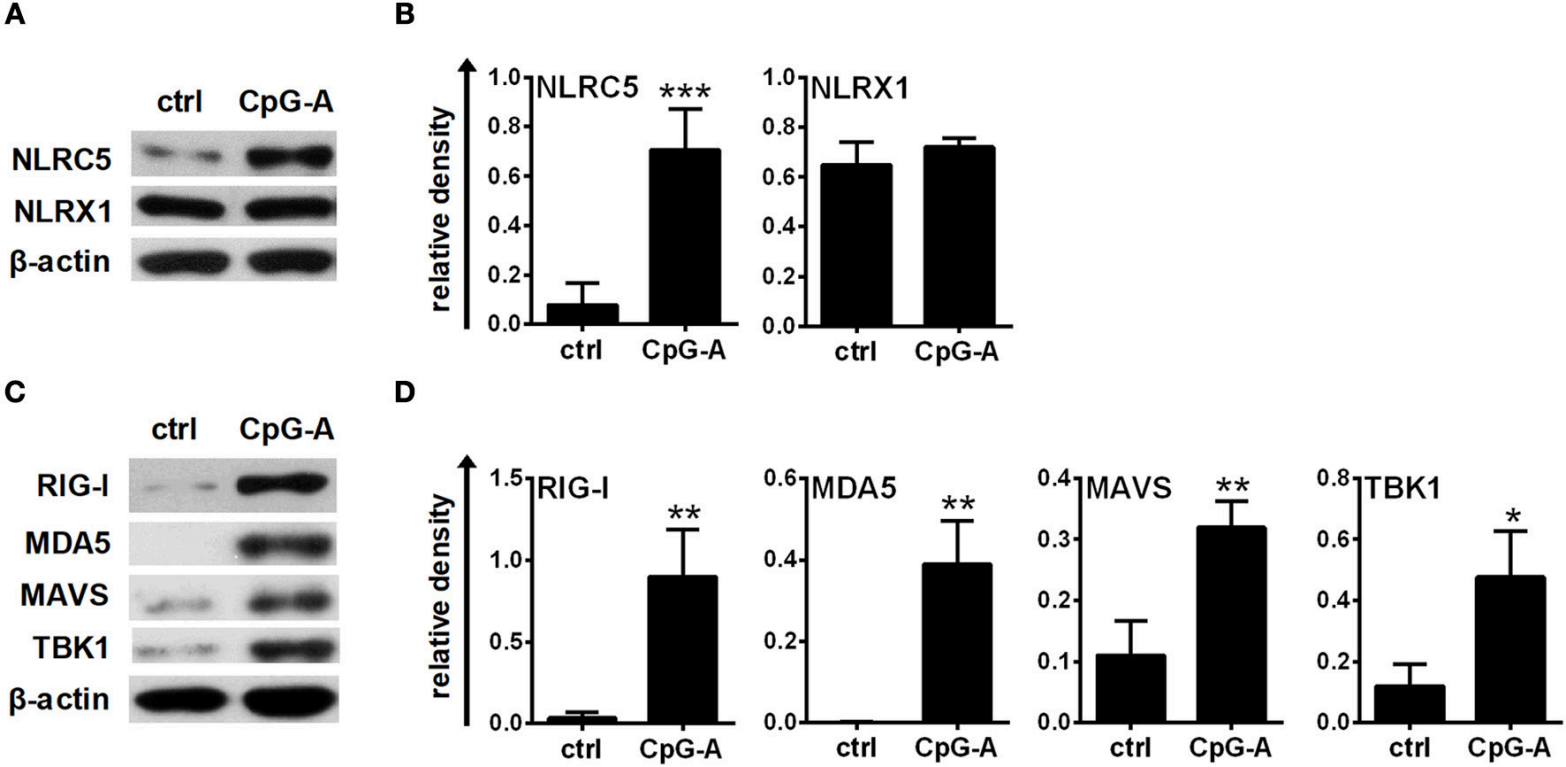
The expression of NLRC5, RIG-I, and MDA5 is inducible in primary human pDCs after CpG-A treatments. **(A–D)** Freshly isolated primary human pDCs were stimulated with 2.5 μM CpG-A for 16 h, thereafter the protein levels of NLRC5, NLRX1, RIG-I, MDA5, MAVS, and TBK1 were detected by western blotting. Representative blots are shown in **(A,C)**. Data are shown as mean ± SD from 3 to 4 experiments in panels **(B,D)**. **(B,D)** Statistical comparisons were performed using Student's *t*-test. ^*^p < 0.05, ^**^p < 0.01, ^***^p < 0.001.

Similar results were obtained using freshly isolated primary human pDCs; namely NLRX1 is constantly expressed whereas NLRC5 is inducible upon CpG-A stimulation (Figures 2A,B). Furthermore, RIG-I, MDA5, MAVS, and TBK1 were all strongly upregulated following CpG-A treatment (Figures 2C,D). Thus, our observations indicate that GEN2.2 cells provide a relevant cell model for studying NLR and RLR interactions in human pDCs.

### Silencing of NLRC5 or NLRX1 does not alter the expression of RLR signaling components in GEN2.2 cells

pDC are best known for their ability to produce high levels of type I IFNs as well as a broad array of pro-inflammatory cytokines in response to many viruses (36). We were curious whether NLRs are able to regulate RIG-I-mediated antiviral responses of pDCs. Therefore we performed siRNA-mediated gene silencing to deplete NLRC5 and NLRX1 in GEN2.2 cells. At 24 h post transfection cells were stimulated with 0.25 μM of CpG-A followed by which the efficacy of gene silencing and the expression of RLR signaling molecules were verified by western blot analysis (Figure S1). Our data show that gene silencing by siRNAs significantly reduced the level of the targeted proteins both in resting and activated cells (>80%; Figures S1A–D). We also assessed RIG-I, MDA5 and MAVS protein levels in order to reveal if depletion of either NLRC5 or NLRX1 impacts their expression. We found that siRNA transfection did not alter the expression pattern of RLR signaling proteins; MAVS is not affected whereas RIG-I and MDA5 are absent from resting GEN2.2 cells but inducible upon CpG-A stimulation regardless of NLRC5 or NLRX1 silencing (Figures S1E–H).

### NLRC5 and NLRX1 inhibit type I IFN responses but not the production of pro-inflammatory cytokines in GEN2.2 cells

**Figure 3:**
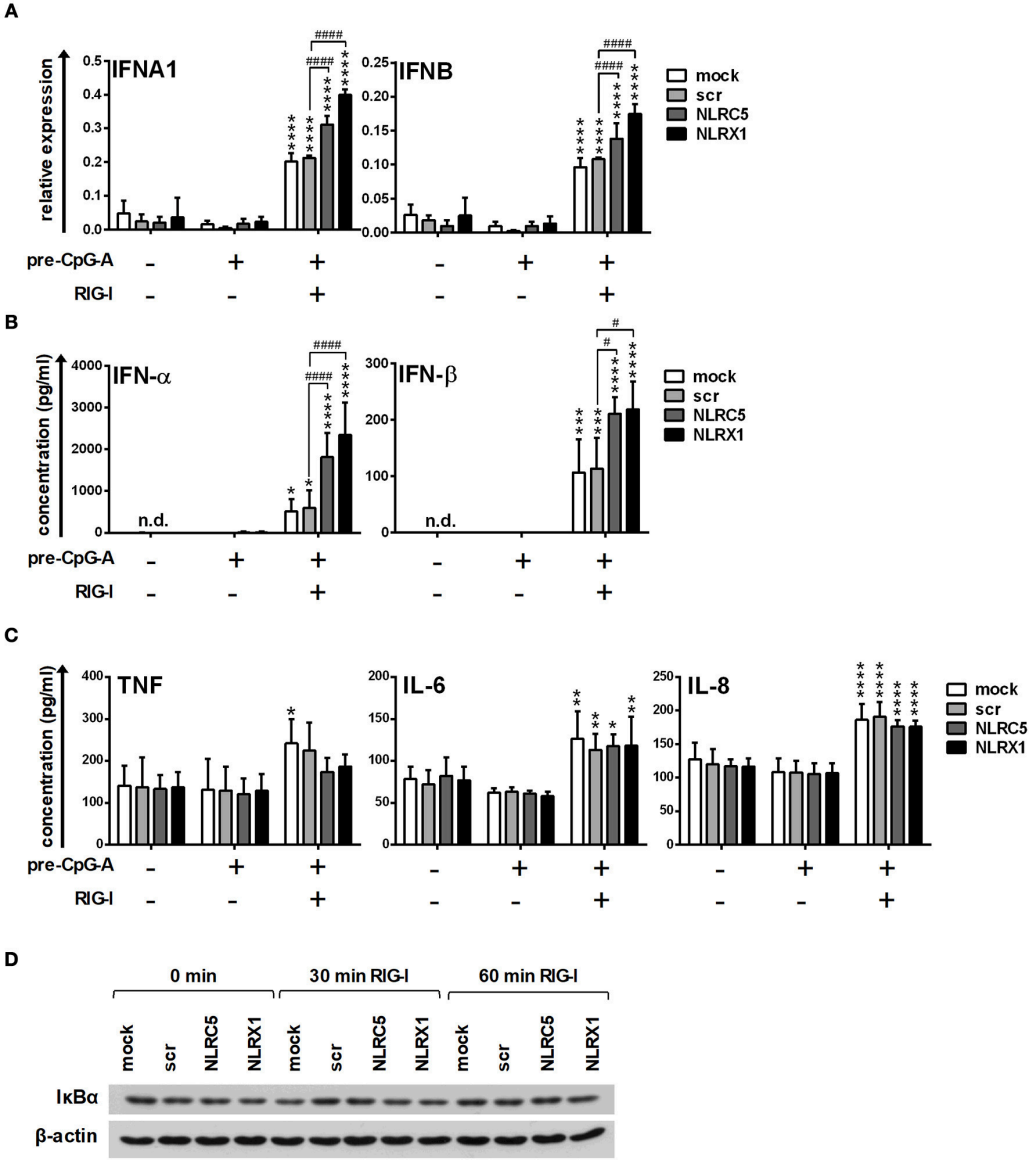
The specific RIG-I agonist-induced type I IFN production is upregulated by NLRC5 or NLRX1 silencing while the NF-κB signaling pathway is not affected in GEN2.2 cells. **(A–D)** Cells were transfected with siRNAs specific for NLRC5, NLRX1 or scrambled (scr) siRNAs for 24 h then pre-treated with 0.25 μM CpG-A (pre-CpG-A) for 16 h to induce the cytosolic expression of RLRs. Following thorough washing steps cells were stimulated with the specific RIG-I agonist 5′ppp-dsRNA (RIGL, 1 μg/ml). The *IFNA1* and *IFNB* mRNA expression levels were assessed by real-time PCR after 3 h **(A)** and IFN-α, IFN-β **(B)**, TNF, IL-6, and IL-8 **(C)** protein levels were measured by ELISA after 6 **(B)** or 24 h **(C)**. **(D)** Kinetics of IκBα degradation was determined by western blotting. **(D)** A representative blot is shown. **(A–C)** Data are represented as means ± SD of 3-5 individual experiments and analyzed using one-way ANOVA followed by Bonferroni's *post-hoc* test. ^*^p < 0.05, ^**^p < 0.01, ^***^p < 0.001, ^****^p < 0.0001 vs. pre-CpG-A-treated samples; ^#^*p* < 0.05, ^####^*p* < 0.0001, n.d., not determined.

Following successful transfection, resting or CpG-A pre-treated cells were activated with the specific RIG-I ligand 5′ppp-dsRNA. Interestingly, NLRC5 and NLRX1 depletion increased the transcript levels of *IFNA1* and *IFNB* (Figure 3A) and elicited a stronger IFN-α and IFN-β production to 5′ppp-dsRNA (Figure 3B). These data demonstrate that both NLRC5 and NLRX1 play negative regulatory roles in the RIG-I-mediated IFN responses of human pDCs.

In addition to IFNs, pDC are also able to rapidly produce pro-inflammatory cytokines and chemokines upon viral infection (36); therefore we analyzed the secretion of the pro-inflammatory cytokines IL-6, TNF, and the chemokine IL-8 protein in the cell culture supernatants by ELISA. As shown in Figure 3C, low levels of the investigated proteins were produced constitutively and could be only slightly increased by RIG-I stimulation in GEN2.2 cells. Interestingly, in contrast to type I IFNs, the secretion of TNF, IL-6 and IL-8 was not affected by NLRC5 or NLRX1 silencing. To further confirm that NLRC5 and NLRX1 do not influence the NF-κB signaling pathway we measured NF-κB activity by investigating the degradation kinetics of its inhibitory protein, IκBα (Figure 3D). Degradation of IκBα releases the p50 and p65 subunits of NF-κB, allowing their nuclear translocation and subsequent activation of target genes. Interestingly, RIG-I stimulation failed to induce IκBα degradation that correlated with the weak pro-inflammatory cytokine production suggesting that RIG-I preferentially induces the production of type I IFNs over pro-inflammatory cytokines in a non-canonical way in pDCs, in accordance with previous reports from animal *in vivo* studies (37).

**Figure 4:**
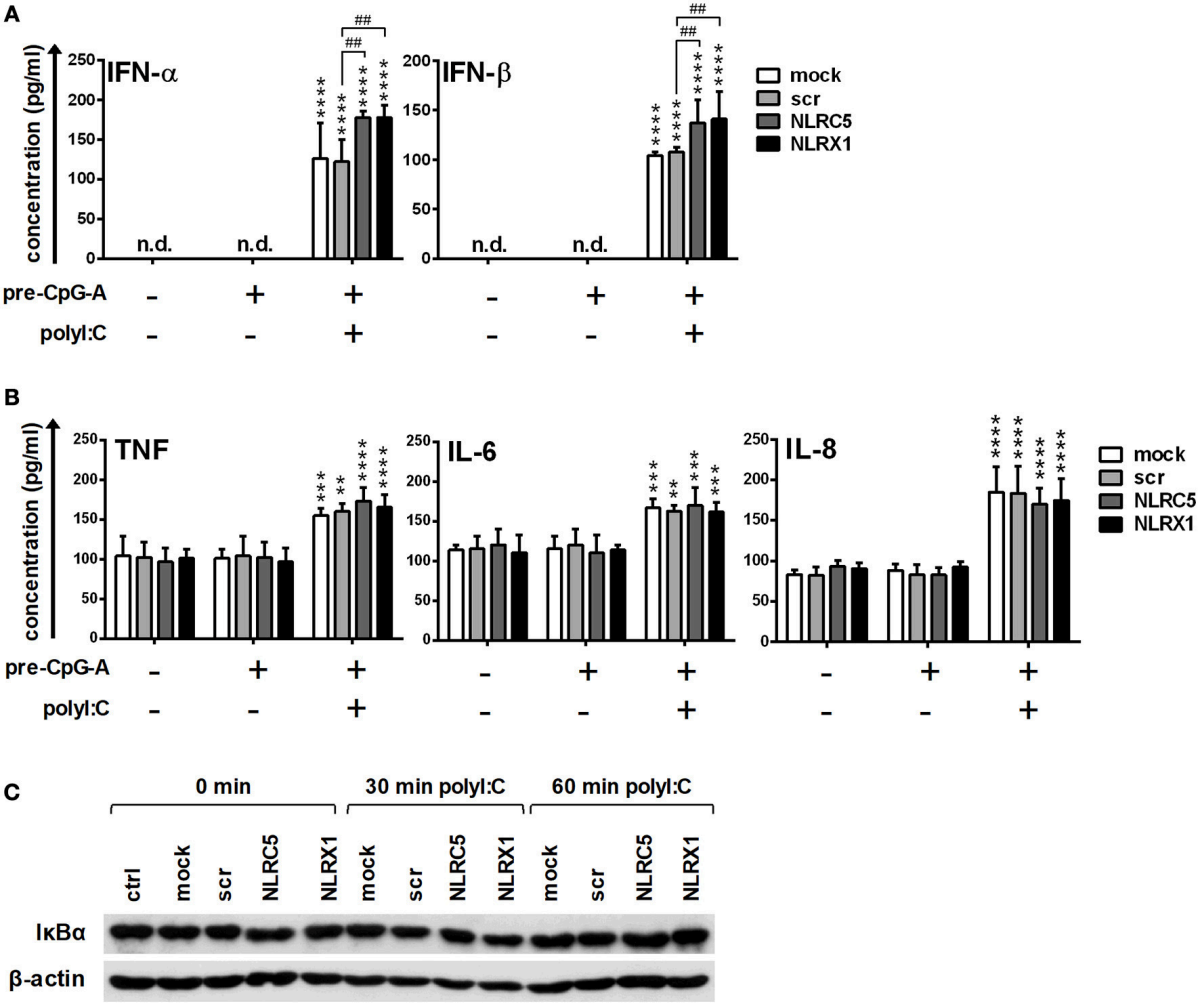
The RIG-I/MDA5 agonist-induced type I IFN production is upregulated by NLRC5 or NLRX1 silencing while the NF-κB signaling pathway is not affected in GEN2.2 cells. **(A–C)** Cells were transfected with siRNAs specific for NLRC5, NLRX1 or scrambled (scr) siRNAs for 24 h then pre-treated with 0.25 μM CpG-A (pre-CpG-A) for 16 h to induce the cytosolic expression of RLRs. Following thorough washing steps cells were stimulated with the RIG-I/MDA5 agonist polyI:C (1 μg/ml). The protein levels of IFN-α, IFN-β (**A**), TNF, IL-6, and IL-8 **(B)** were measured by ELISA after 6 **(A)** or 24 h **(B)**. **(C)** Kinetics of IκBα degradation was determined by western blotting. **(C)** A representative blot is shown. Data are represented as means ± SD of 4 individual experiments and analyzed using one-way ANOVA followed by Bonferroni's *post-hoc* test. ^**^p < 0.01, ^***^p < 0.0001, ^****^p < 0.0001 vs. pre-CpG-A-treated samples; ^##^*p* < 0.01, n.d., not determined.

In separate experiments, following CpG-A pre-treatment, cells were re-stimulated with polyinosinic:polycytidylic acid, (polyI:C) instead of the specific RIG-I agonist 5′ppp-dsRNA (Figure 4). PolyI:C is a ligand for both cytoplasmic RIG-I/MDA5 and endosomal TLR3 as well. Whereas naked polyI:C is recognized by TLR3, transfected polyI:C is sensed by RIG-I/MDA5 (20). In our experiments we used polyI:C-HMW/LyoVec, a complex between high molecular weight polyI:C and the transfection reagent LyoVec, which signals only through RLRs. Although, LyoVec-conjugated polyI:C-HMW is an agonist both for RIG-I and MDA5, studies have reported that it is preferentially recognized by MDA5 (14, 38). We obtained very similar results when polyI:C/LyoVec was used as a stimulant. NLRC5 and NLRX1 depletion increased the polyI:C-mediated secretion of type I IFNs (Figure 4A) but did not affect the production of pro-inflammatory cytokines (Figure 4B) and NF-κB activity (Figure 4C).

To further test the assumption that NLRC5 and NLRX1 does not regulate the pro-inflammatory cytokine production in pDCs we stimulated GEN2.2 cells with well-known stimulators of the NF-κB signaling pathway including the TLR9 ligand CpG-B, the TLR7 ligand imiquimod and the TLR1/2 receptor agonist PAM3CSK4 (Figure S2A). The kinetics of IκBα degradation revealed that CpG-B and imiquimod are strong inducers of the NF-κB signaling route; therefore, we used these agonists to induce a pro-inflammatory signal following depletion of NLRC5 and NLRX1. Our results demonstrate that neither NLRC5 nor NLRX1 silencing has any effect on the TNF, IL-6 and IL-8 cytokine production upon CpG-B or imiquimod stimulation (Figure S2B). These results confirm our observation that the NF-κB signaling route is indeed not affected by NLRC5 and NLRX1.

Collectively, our results indicate that NLRC5 and NLRX1 play a critical regulatory role in the RLR-mediated type I IFN responses while not affecting NF-κB activity, and thus the pro-inflammatory cytokine production of human pDCs.

### Immature moDCs constantly express NLRC5 and NLRX1, the silencing of which does not affect their differentiation and the expression of RLR signaling components

Various DC subtypes show distinct inflammatory cytokine profiles under steady-state and inflammatory conditions (39). In order to investigate how NLRX1 and NLRC5 affect the RIG-I mediated immune responses of other DC subsets, we repeated our experiments with moDCs. Under inflammatory conditions, monocytes can differentiate into moDCs, which in response to microbial stimuli are able to produce large amounts of pro-inflammatory and/or antiviral cytokines thus contribute to the shaping of innate and adaptive immune responses (40–42). Therefore, moDCs generated from CD14^+^ blood monocytes in the presence of GM-CSF and IL-4 *in vitro* serve as an excellent model for studying DC functionality.

**Figure 5:**
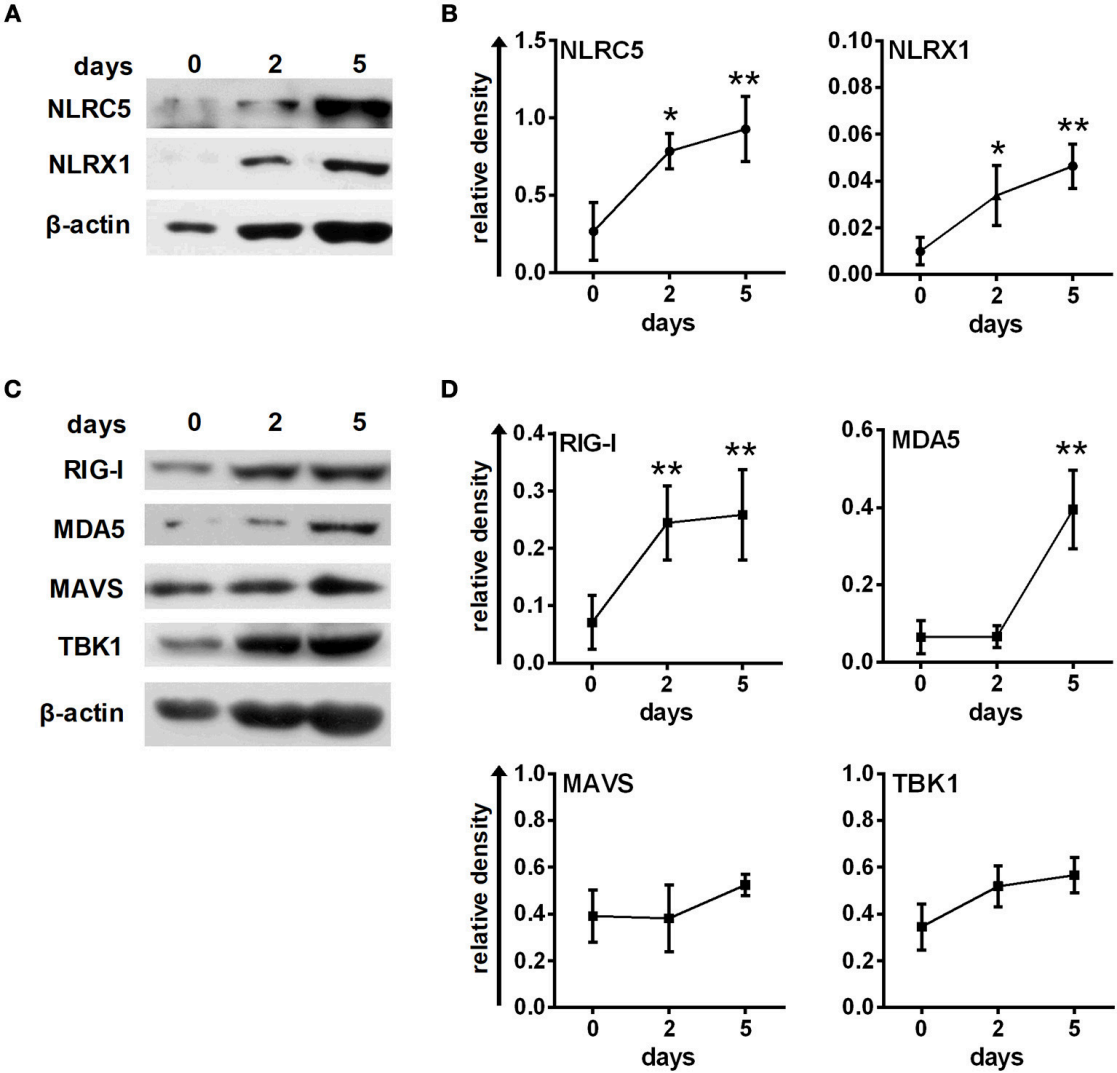
Human immature moDCs constantly express NLRC5, NLRX1, RIG-I and MDA5. **(A–D)** Freshly isolated monocytes were seeded in 24-well plates and differentiated as described in the “Materials and Methods.” The protein levels of NLRC5, NLRX1, RIG-I, MDA5, MAVS, and TBK1 were measured by western blot. **(A,C)** Representative blots are shown. **(B,D)** Graphs represent the kinetics of protein expressions during moDC differentiation. Data are represented as mean ± SD of 3–5 individual experiments and were analyzed using one-way ANOVA followed by Bonferroni's *post-hoc* test. ^*^p < 0.05, ^**^p < 0.01 vs. day 0.

First, we analyzed the expression profile of NLRC5, NLRX1, and also RLR signaling molecules during moDC differentiation as it is poorly characterized so far in human cells (Figure 5). We found that NLRC5, NLRX1 are either not detectable or only weakly expressed in freshly isolated monocytes but are gradually upregulated during moDC differentiation (Figures 5A,B). Similarly, RIG-I, MDA5 and their downstream signaling components including MAVS and TBK1 are all upregulated during the DC differentiation process (Figures 5C,D).

In the next step, we performed RNA interference on freshly isolated monocytes using a method we previously applied successfully to silence target genes in moDCs (43). Monocytes were transfected with siRNAs specific for NLRC5 and NLRX1, scrambled control siRNA as described in “Materials and Methods” then the efficiency of gene silencing was evaluated by western blot analysis on day 5 of moDC differentiation (Figures S3A,B). Our data demonstrate a successful downregulation of the target genes that does not affect the expression of RIG-I, MDA5, or MAVS in immature moDCs (Figures S3C,D).

**Figure 6:**
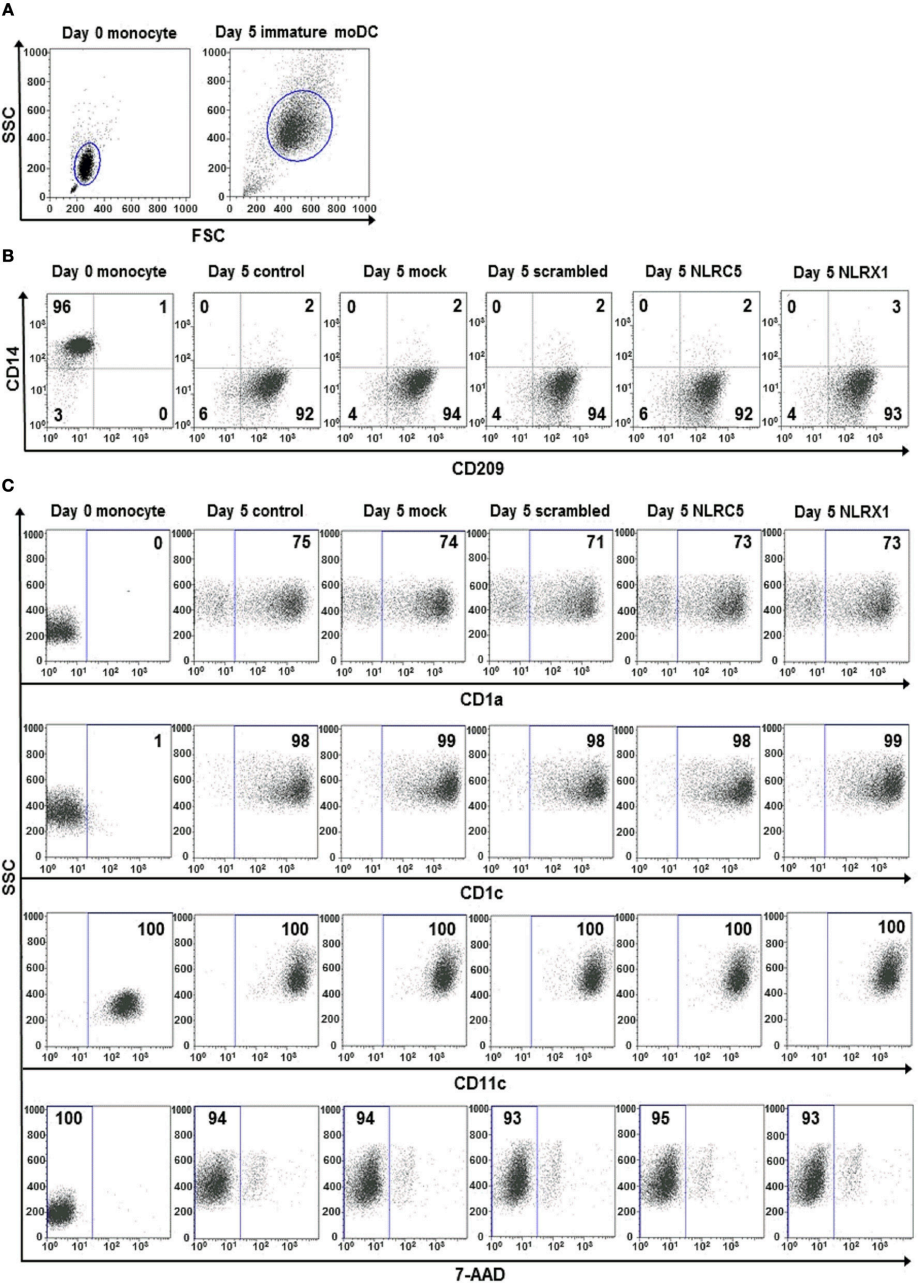
The silencing of NLRC5 or NLRX1 does not influence the differentiation process of human immature moDCs. **(A–C)** Freshly isolated monocytes were transfected with siRNAs specific for NLRC5, NLRX1, or scrambled (scr) siRNAs at day 0 and differentiated into immature moDCs. On day 5 of differentiation the phenotypic analysis of the cells were perfomed by flow cytometry. Cells were gated on forward vs. side scatter to exclude debris **(A)** and the expression levels of CD14 and CD209 **(B)** as well as CD1a, CD1c, and CD11c cell surface proteins and cell viability **(C)** were analyzed. **(A–C)** Representative dot blots are shown from 3 individual experiments. **(B, C)** Isotype controls antibodies were used to set gates for positive events and numbers indicate the percentage of positive cells. In case of 7AAD staining the numbers show the ratio of 7-AAD negative live cells.

To confirm that siRNA silencing does not interfere with moDC differentiation we measured the expression of the monocyte-specific marker, CD14, and DC-specific ICAM-3-grabbing nonintegrin (CD209/DC-SIGN; Figure 6). Our results show that the CD209/CD14 ratio increased at a comparable level in cells transfected with siRNAs as compared with untreated cells on day 5 of moDC differentiation, indicating that inhibition of NLRC5 or NLRX1 does not influence the differentiation processes of moDCs (Figure 6B). A profound day-to-day analysis of CD209/CD14 ratio further confirmed that neither NLRC5 nor NLRX1 silencing influence the differentiation process of monocytes into DCs (Figure S4). To further identify the phenotype of differentiated moDC we also measured the expression of surface markers characteristic of DCs including CD1a, CD1c, and CD11c (Figure 6C). Our results demonstrate that all DC specific markers are present in a high percentage (CD1a: >71 %, CD1c: >98%, CD11c: 100%) on the surface of immature moDCs regardless of NLRC5 and NLRX1 silencing. We also assessed cell viability by using 7-AAD single staining and found that it is not affected by siRNA transfection (Figure 6C). To test whether NLRC5 and NLRX1 silencing induce unintended maturation of moDCs, we also screened 5-day moDCs for co-stimulatory molecules and maturation markers including CD40, CD80, CD83 and the MHC class II molecule HLA-DR (Figure S5). Our results indicate that compared to the untreated or scrambled siRNA-transfected cells, silencing with siRNAs specific for NLRC5 and NLRX1 did not affect the expression of maturation markers, suggesting that the cells remained in their immature state regardless of NLR depletion.

### RLR-mediated antiviral and pro-inflammatory responses are affected by NLRX1 but not by NLRC5 in moDCs

**Figure 7:**
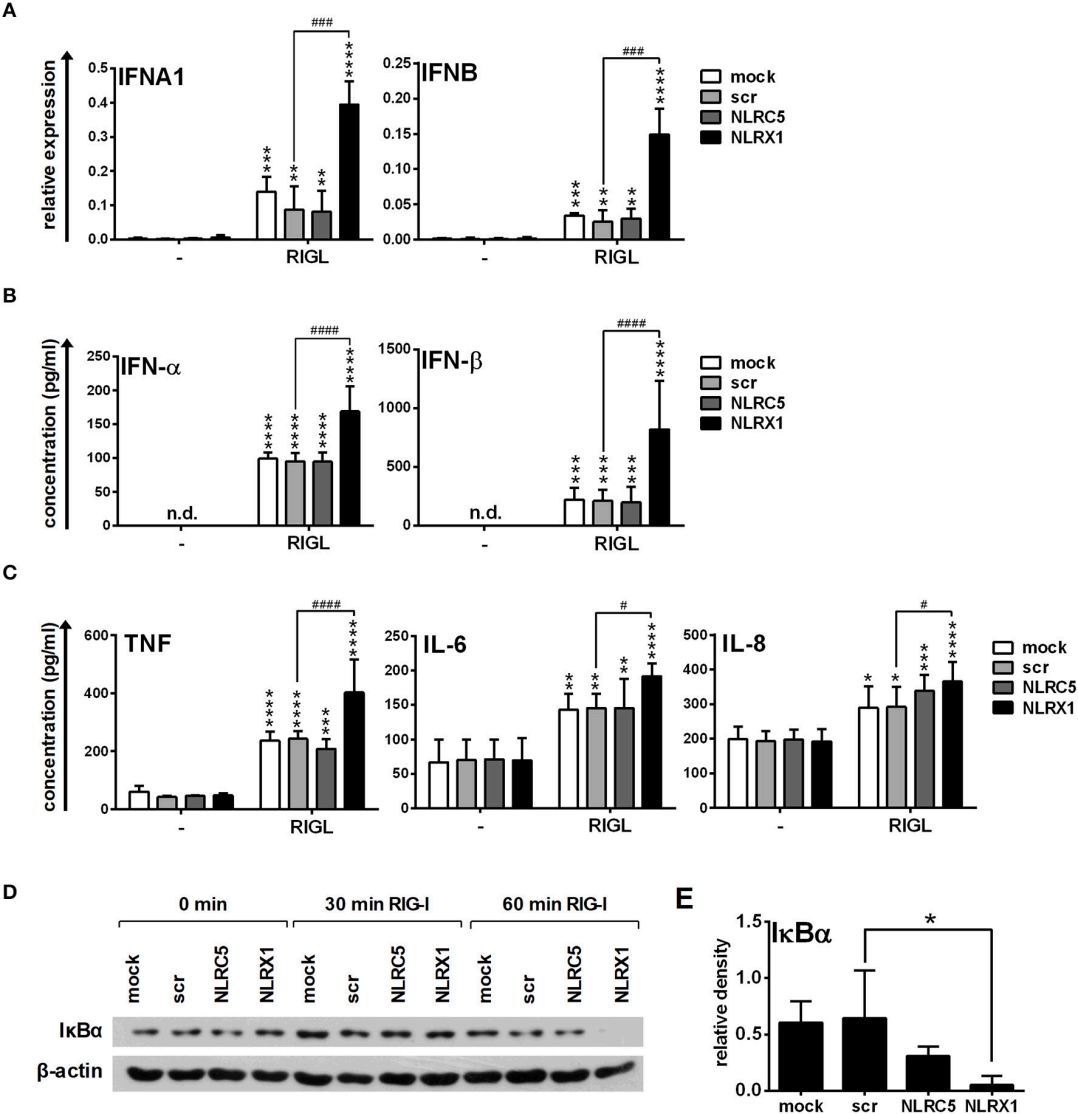
NLRX1 but not NLRC5 affects the specific RIG-I agonist-induced type I IFN and pro-inflammatory responses in human moDCs. **(A–E)** moDCs transfected with the indicated siRNAs were stimulated with the RIG-I ligand 5′ppp-dsRNA (RIGL, 1 μg/ml). The mRNA expression levels of *IFNA1* and *IFNB* were assessed by real-time PCR after 12 h **(A)** and IFN-α, IFN-β **(B)**, TNF, IL-6, and IL-8 **(C**) protein levels were measured by ELISA after 24 h. **(D,E)** Kinetics of IκBα degradation was determined by western blotting. **(D)** A representative blot is shown. **(E)** Bar graphs show the relative density of IκBα measured at 60 min of stimulation. **(A-C, E)** Data are shown as mean ± SD from 4 independent experiments and analyzed using one-way ANOVA followed by Bonferroni's *post-hoc* test. ^*^p < 0.05, ^**^p < 0.01, ^***^p < 0.01 ^****^p < 0.0001 vs. untreated; ^#^*p* < 0.05, ^###^*p* < 0.001, ^####^*p* < 0.0001, n.d., not determined.

Following transfection, resting moDCs were stimulated with the RIG-I specific ligand 5′ppp-dsRNA on day 5 and the antiviral (Figures 7A,B) and pro-inflammatory cytokine profile (Figure 7C) were analyzed. First we measured the expression of IFN-α and IFNβ, the major type I IFNs produced by moDCs. Interestingly, the RIG-I-mediated type I IFN expression is not affected by NLRC5 silencing (Figures 7A,B), whereas it is upregulated by NLRX1 silencing both at the mRNA and protein levels (Figures 7A,B). TNF, IL-6 and IL-8 are secreted at relatively low levels in immature moDCs but a significant increase could be elicited by RIG-I stimulation (Figure 7C). Similarly to GEN2.2 cells, the production of TNF, IL-6, and IL-8 is not influenced by NLRC5 silencing in moDCs. On the contrary, secretion of these cytokines is significantly increased in cells silenced with NLRX1 siRNAs as compared to the scrambled siRNA-transfected cells. The increased production of the pro-inflammatory cytokines suggests elevated NF-κB activity. Measuring the protein levels of IκBα, we have indeed observed degradation at 60 min of stimulation with 5′ppp-dsRNA (Figures 7D,E).

**Figure 8:**
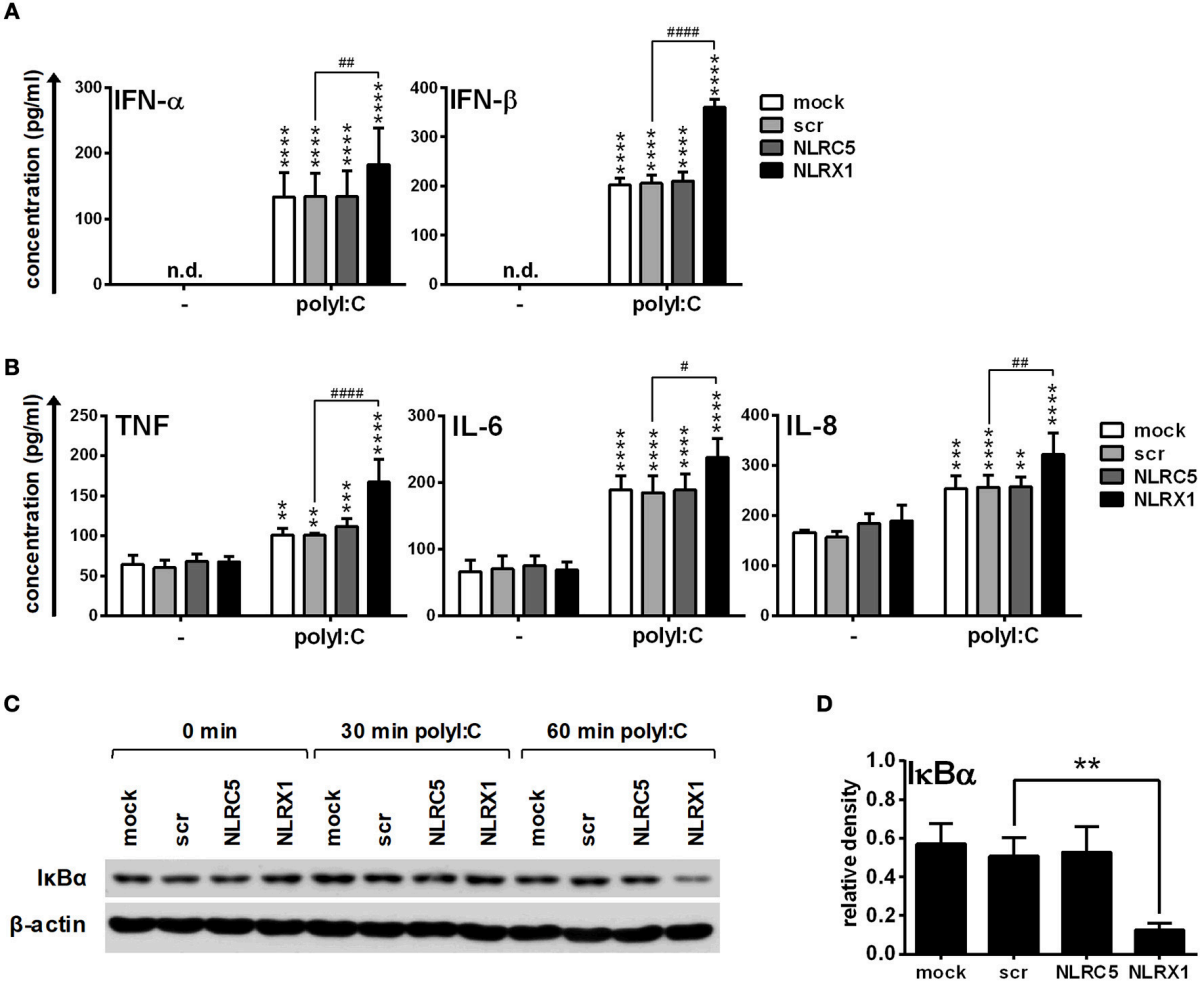
NLRX1 but not NLRC5 controls the RIG-I/MDA5 agonist-induced type I IFN and pro-inflammatory responses in human moDCs. **(A–D)** moDCs transfected with the indicated siRNAs were stimulated with the RIG-I/MDA5 ligand polyI:C (1 μg/ml). The protein levels of IFN-α, IFN-β **(A)**, TNF, IL-6, and IL-8 **(B)** were detected by ELISA after 24 h. **(C,D)** Kinetics of IκBα degradation was determined by western blotting. **(C)** A representative blot is shown. **(D)** Bar graphs show the relative density of IκBα measured at 60 min of stimulation. **(A,B,D)** Data are shown as mean ± SD from 4 independent experiments and analyzed using one-way ANOVA followed by Bonferroni's *post-hoc* test. ^**^p < 0.01, ^***^p < 0.01 ^****^p < 0.0001 vs. untreated; ^#^*p* < 0.05, ^##^*p* < 0.01, ^####^*p* < 0.0001, n.d., not determined.

Similarly to GEN2.2 cells we also stimulated moDCs with polyI:C-HMW/LyoVec that induced the production of type I IFNs and pro-inflammatory cytokines in a manner comparable to 5′ppp-dsRNA (Figure 8). NLRX1 silencing upregulated whereas NLRC5 depletion did not affect the type I IFN and pro-inflammatory cytokine production of moDCs (Figures 8A,B). Furthermore, polyI:C-HMW/LyoVec induced IκBα degradation at a similar rate as 5′ppp-dsRNA at 60 min of stimulation (Figures 8C,D).

Taken together, our results indicate that NLRX1 negatively regulates both interferon and pro-inflammatory responses initiated by RLRs, whereas NLRC5 does not seem to play any regulatory role in these processes in moDCs.

### NLRC5 and NLRX1 mediate antiviral and pro-inflammatory responses to live virus infection of human DCs

**Figure 9:**
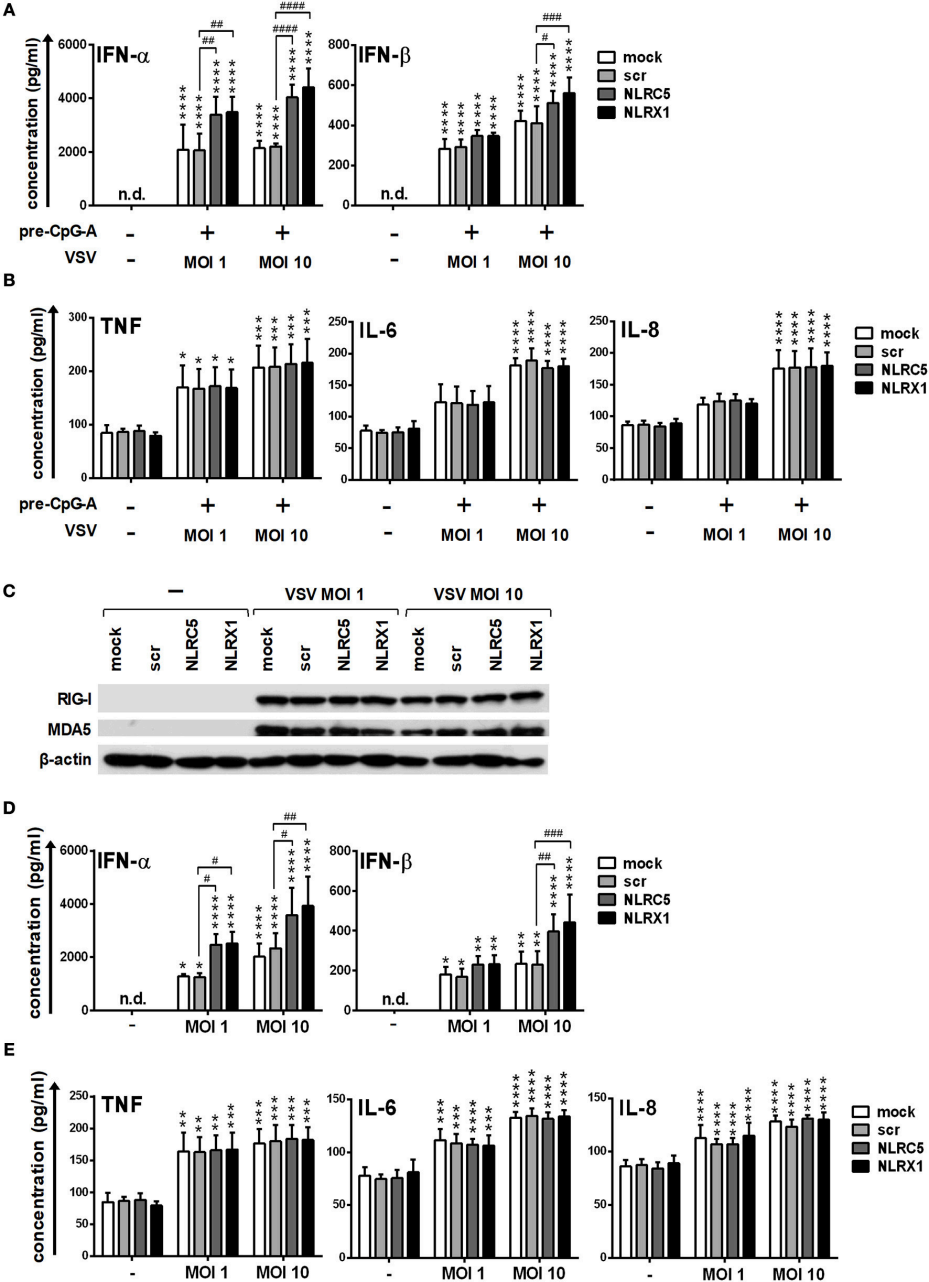
Depletion of NLRC5 or NLRX1 enhances the type I IFN production of GEN2.2 cells but does not influence the NF-κB pathway activity in response to VSV infection. **(A–E)** Cells were transfected with siRNAs specific for NLRC5, NLRX1 or scrambled (scr) siRNAs for 24 h. **(A,B)** After silencing cells were pre-treated with 0.25 μM CpG-A (pre-CpG-A) for 16 h to induce the cytosolic expression of RLRs. Following thorough washing steps cells were infected with VSV at the indicated MOIs. The protein levels of IFN-α, IFN-β **(A)**, TNF, IL-6, and IL-8 **(B)** were measured by ELISA after 18 h. **(C–E)** After silencing GEN2.2 cells were exposed to VSV at the indicated MOIs without CpG-A pre-treatment and the protein levels of RIG-I and MDA5 were detected by western blot at 24 h **(C)**. Concentrations of IFN-α, IFN-β **(D)**, TNF, IL-6, and IL-8 **(E)** were measured by ELISA from the supernatant of the VSV-infected cells. **(C)** A representative blot is shown. Data are represented as means ± SD of 4 individual experiments and analyzed using one-way ANOVA followed by Bonferroni's *post-hoc* test. ^*^p < 0.05, ^**^p < 0.01, ^***^p < 0.0001, ^****^p < 0.0001 vs. untreated; ^#^*p* < 0.05, ^##^*p* < 0.01, ^###^*p* < 0.001, ^####^*p* < 0.0001, n.d., not determined.

To strengthen our observations we also carried out experiments with live virus both in GEN2.2 cells and moDCs. Following transfection, CpG-A pre-treated cells were activated with VSV at a MOI of 1 or 10 for 18 h. Compared to the synthetic ligands 5′ppp-dsRNA and polyI:C, VSV-induced type I IFN production was increased both by NLRC5 and NLRX1 silencing (Figure 9A), whereas the pro-inflammatory cytokine production was not affected (Figure 9B).

We also wanted to investigate whether VSV is able to upregulate the expression of RLRs similarly to the CpG-A treatment. Our results indicate that RIG-I and MDA5 are strongly induced upon VSV infection (Figure 9C). These observations prompted us to analyze the VSV-induced cytokine responses of NLRC5- and NLRX1-depleted GEN2.2 cells that were not pre-treated with CpG-A. We found that NLRC5 and NLRX1 silencing resulted in similar cytokine profile of VSV-infected GEN2.2 cells independently of CpG-A pre-treatment (Figures 9D,E).

**Figure 10:**
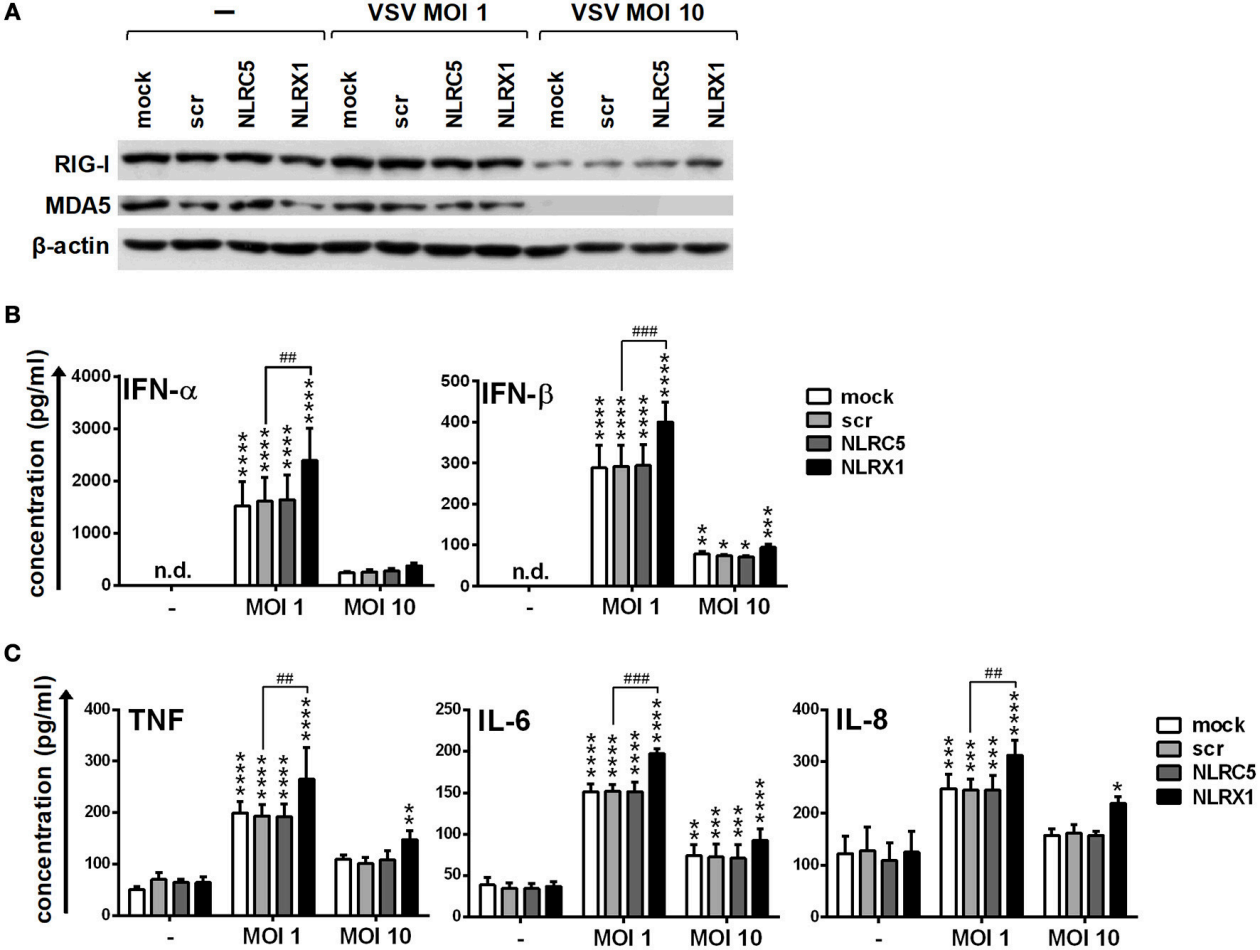
NLRX1 but not NLRC5 affects the type I IFN and pro-inflammatory responses in VSV-infected human moDCs. **(A–C)** moDCs transfected with the indicated siRNAs were exposed to VSV at the indicated MOIs and after 18 h the protein levels of RIG-I and MDA5 **(A)** were analyzed by western blotting, and the concentrations of secreted IFN-α, IFN-β **(B)**, TNF, IL-6, and IL-8 **(C)** were determined by ELISA. **(A)** A representative blot is shown. **(B,C)** Data are shown as mean ± SD from 4 independent experiments and analyzed using one-way ANOVA followed by Bonferroni's *post-hoc* test. ^*^p < 0.05, ^**^p < 0.01, ^***^p < 0.01 ^****^p < 0.0001 vs. untreated; ^##^*p* < 0.01, ^###^*p* < 0.001, n.d., not determined.

Repeating our experiments with moDCs we found that the expression of RIG-I and MDA5 is not affected by infection with VSV at a MOI of 1 whereas strongly decreased at a MOI of 10 (Figure 10A). Compared to the synthetic ligands 5′ppp-dsRNA and polyI:C, VSV at a MOI of 1 induced type I IFN and pro-inflammatory cytokine production that was increased upon NLRX1 depletion but not affected by NLRC5 silencing (Figures 10B,C). Interestingly, in contrast to GEN2.2 cells, moDCs were much more sensitive to virus infection as VSV at a MOI of 10 failed to elicit increased cytokine production, which might be explained by the decreased expression of RIG-I and MDA5 caused by VSV infection.

All these data demonstrate that NLRX1 and NLRC5 can control the type I IFN and pro-inflammatory responses to live virus infection of human DCs as well.

## Discussion

During the course of infections a fundamental role of the host immune system is to induce a rapid and robust immune response to eradicate invading pathogens and then resolve inflammation to restore tissue homeostasis and to induce regenerative processes. A broad range of PRRs are responsible for initiating signaling cascades that mediate the innate immune response following viral infections (1). RLRs recognize viral nucleic acids in the cytosol and signal through MAVS to initiate downstream effector molecules such as type I IFNs and other pro-inflammatory cytokines that serve to mount a local antiviral response (6, 10). Although activation of RLR signaling is necessary to limit the spread of viruses, stringent regulation is needed to prevent excessive immune response that may result in tissue damage and further detrimental antiviral effects on the host (44). RLR-mediated innate immune signaling has been found to be regulated by several regulatory molecules that exert their function both in the steady state and upon viral infection (45). It has been recently recognized that tripartite motif containing 29 (TRIM29) plays a negative regulatory role in type I IFN production in response to polyI:C or dsRNA virus infection in bone marrow–derived DCs and macrophages as well as in response to 5′ppp-RNA in murine alveolar macrophages (46, 47). Furthermore, several members of the NLR family have been also proposed to serve as checkpoint of immune activation, with NLRC5 and NLRX1 directly interacting with the RIG-I mediated antiviral signaling (4, 29).

NLRX1 was initially characterized as a negative regulator of antiviral responses (48). Moore and colleagues described that NLRX1 localizes to the mitochondrial outer membrane where it interacts with MAVS and results in the attenuation of RLR signaling pathways in human epithelial HEK293T cells (48). Since then, several studies have examined the importance of NLRX1 during antiviral signaling both *in vitro* and *in vivo*, but have produced conflicting results and the role of NLRX1 remained controversial. Similarly to the initial report, Allen *et al*. demonstrated that NLRX1^−/−^ mice exhibit increased expression of antiviral signaling molecules such as IFN-β and IL-6 following exposure to viruses, which activate RIG-I (49). In contrast, another group reported that NLRX1 deficiency does not affect RLR/MAVS signaling (50). In a subsequent study, normal antiviral and inflammatory responses have been observed following Sendai virus infection of bone marrow-derived macrophages and murine embryonic fibroblasts from NLRX1-deficient mice (51). Furthermore, an independent study reported that NLRX1-silenced HEK293T cells infected with Sendai virus displayed normal type I IFN response (52). Here we show that NLRX1 is constitutively expressed both in human pDCs and moDCs and interferes with the RIG-I-mediated type I IFN production in these cell types. So far one study has proposed a role for NLRX1 in human DCs, showing that NLRX1 promotes HIV-1 infection in multiple cell types including human primary macrophages and DCs (53). We found that NLRX1 silencing significantly increased pro-inflammatory cytokine (TNF, IL-6) and chemokine (IL-8) secretion in moDCs but not in pDCs following RLR stimulation. These findings were further supported by the observation that IκBα is degraded only in NLRX1-depleted moDCs upon stimulation with the RIG-I ligand 5′ppp-dsRNA or the RIG-I/MDA5 agonist polyI:C. Thus, our results indicate that NLRX1 predominantly acts as a negative regulator of RLR/MAVS signaling in human DCs.

NLRC5 is primarily known as an MHC class I transactivator, which following activation localizes to the nucleus and induces the promoters of MHC class I genes (54). NLRC5 is widely expressed in hematopoietic cells and can be greatly induced by type I and II IFNs produced in response to pathogenic infections (55). Consistent with these observations, we demonstrate that NLRC5 is gradually upregulated in differentiating moDC and upon TLR9 activation in pDCs.

Increasing evidence indicates that NLRC5 contributes to innate and adaptive immune responses, though the published studies present apparently conflicting results. In the context of virus infection NLRC5 was initially reported to be a positive regulator of type I IFN responses (56). The authors show that siRNA-mediated silencing of NLRC5 impairs the upregulation of IFN-α after human cytomegalovirus infection of human foreskin fibroblasts (56). Similar results on the function of NLRC5 were reported by another group demonstrating that knockdown of endogenous NLRC5 impairs type I IFN responses in THP1 cells and human primary dermal fibroblast in response to Sendai virus and polyI:C (57). By contrast, another group showed that NLRC5 negatively regulates the NF-κB and type I IFN pathways by direct interaction with IκB kinase (IKK) and RIG-I in multiple cell lines and primary cells (58). In detail, following viral infection or stimulation by specific ligands, the caspase recruitment domain (CARD) of RIG-I and MDA5 becomes accessible for NLRC5 which competes with MAVS for binding, thus leading to dampened IRF3 activation (58). In a subsequent study the same group generated NLRC5 knockout mice and found that NLRC5 deficiency increased IL-6 and IFN-β production in mouse embryonic fibroblast, peritoneal and bone marrow-derived macrophages but not in bone marrow-derived DCs following vesicular stomatitis virus infection or lipopolysaccharide stimulation (59). Interestingly, a study by Kumar *et al*. suggests that NLRC5 is dispensable for cytokine induction in bone marrow-derived macrophages and DCs in response to a range of viral and bacterial infections (60). The authors also published that NLRC5 directly binds to TBK1 and suppresses TBK1-mediated IFN-β promoter activation in HEK293T cells (60). Our data demonstrate that NLRC5 acts as a negative regulator of type I IFN production of pDCs but not of moDCs. Investigating the role of NLRC5 in RLR-mediated pro-inflammatory signaling we could not detect any differences between control and NLRC5-depleted pDCs and moDCs. Altogether, our results suggest that NLRC5 modulates RLR-mediated type I IFN responses but not the pro-inflammatory signaling of pDCs and seems to play no role in the control of RIG-I signaling pathway dynamics in moDCs.

Finally, to confirm our findings both pDC cell line and moDCs were infected with live, replicating viruses. Since there are not any virus strains that are exclusively recognized by RIG-I or MDA5, we decided to use VSV, a single-stranded negative-sense RNA virus in our experiments. VSV is preferentially recognized by RLRs, but can also be detected by TLR7 (9, 61). The results obtained by virus infection were consistent to those observed using the synthetic ligands 5′ppp-dsRNA and polyI:C both in GEN2.2 cells and moDCs. Further, we tested whether VSV is able to induce RLR expression in GEN2.2 cells and found that both RIG-I and MDA5 are significantly upregulated after 24 h of infection. Previously, we published that both endosomal TLR7 and TLR9 are able to upregulate RIG-I expression in human pDCs (26); thus, we assume that the initial recognition and triggering of RLR expression might be mediated by TLR7. In contrast to pDCs, moDCs were more sensitive to infection with VSV. At high viral dose, we observed degradation of RIG-I and MDA5 proteins that consequently lead to lower cytokine production. It is known that pDCs possess faster and stronger IFN producing abilities compared to other cell types including moDCs (62). Our results also indicate that pDCs are better equipped with antiviral proteins that exert a protective effect against the cytopathic effects of VSV.

Recently several studies have tried to unfold the role of regulatory NLRs in antiviral signaling pathways, however yielded inconsistent findings. The discrepancies can be attributed to several factors such as the different cell types/lines and silencing/overexpression techniques used in each study that might greatly influence the results. Several of the aforementioned studies applied luciferase reporter assays to measure the activity of signal transduction pathways; however, a recent study has indicated that this technique is not suitable to analyze the role of NLRs due to the presence of leucine rich repeat domain that induces nonspecific aggregation and degradation of luciferase and leads to misinterpretation of experimental data (52). To overcome these limitations, we used siRNA-based silencing technique in our experiments and achieved substantial depletion of NLRC5 and NLRX1 proteins. Our data obtained from transfection experiments are in line with the reports of Allen et al. and Cui et al. (49, 58) indicating that the RLR-mediated antiviral responses are negatively regulated by NLRX1 and at least partially by NLRC5. All these observations including ours support the idea that NLRC5 and NLRX1 as regulatory NLRs play a physiologically important role in the maintenance of immune homeostasis, especially in the modulation of innate immune responses.

Accumulating evidence suggest that aberrant IFN production due to abnormal RLR activation is associated with the development of autoimmune diseases (7). Therefore, understanding the molecular mechanisms underlying the negative regulation of innate immunity might contribute to the development of effective therapies for inflammation-induced autoimmune diseases. From another aspect, these regulatory NLRs working as molecular breaks on antiviral signaling might serve as potential therapeutic targets for enhancing host responses to pathogenic infection.

## Ethics statement

Human buffy coats were obtained from healthy blood donors drawn at the Regional Blood Center of Hungarian National Blood Transfusion Service (Debrecen, Hungary) in accordance with the written approval of the Director of the National Blood Transfusion Service and the Regional and Institutional Ethics Committee of the University of Debrecen, Faculty of Medicine (Debrecen, Hungary). Written informed consent was obtained from the donors prior blood donation, and their data were processed and stored according to the directives of the Declaration of Helsinki.

## Author contributions

KP and TF designed the research, performed experiments, analyzed, and interpreted data and wrote the manuscript. DB and AS performed experiments. EC performed viral studies and participated in data analysis. KP, AB, and TB contributed with essential reagents. All authors reviewed and approved the manuscript.

### Conflict of interest statement

The authors declare that the research was conducted in the absence of any commercial or financial relationships that could be construed as a potential conflict of interest.

## Abbreviations

Cdc: conventional DC
DC: dendritic cell
IFN: interferon
MAVS: mitochondrial antiviral-signaling protein
MDA5: Melanoma differentiation-associated gene-5
moDC: monocyte-derived DC
NF-κB: nuclear factor-κB
NLR: nucleotide-binding domain leucine-rich repeat
pDC: plasmacytoid DC
polyI:C: polyinosinic:polycytidylic acid
PRR: pattern recognition receptor
RIG-I: retinoic-acid inducible gene I
RLR: RIG-I-like receptors
TLR: Toll-like receptors
VSV: Vesicular Stomatitis Virus.
