# Supplementary material for: Regulatory NLRs Control the RLR-Mediated Type I Interferon and Inflammatory Responses in Human Dendritic Cells
Source: Front Immunol. 2018 Oct 5;9:2314. doi: 10.3389/fimmu.2018.02314 (PMC6182093; doi:10.3389/fimmu.2018.02314)
Supplement: Supplementary file 1 [file Data_Sheet_1.docx]

Supplementary Material

**Regulatory NLRs control the RLR-mediated type I interferon and inflammatory responses in human dendritic cell**

**Tünde Fekete, Dora Bencze, Attila Szabo, Eszter Csoma, Tamas Biro, Attila Bacsi, Kitti Pazmandi***

* **Correspondence:** Kitti Pazmandi: [pazmandikitti@yahoo.de](mailto:pazmandikitti@yahoo.de)

## Supplementary Figures


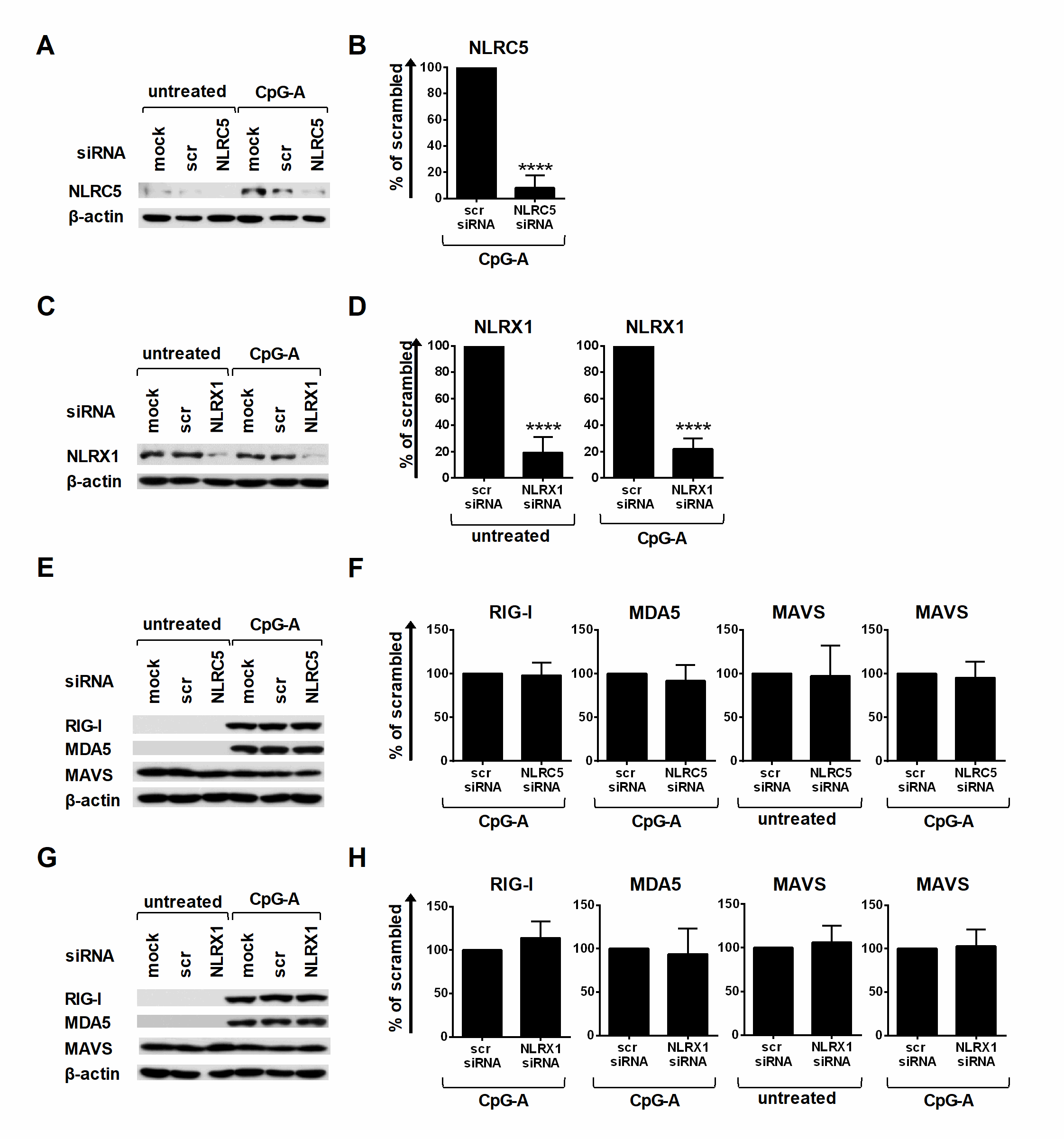


**Supplementary Figure 1.** Silencing of NLRC5 or NLRX1 does not influence the protein level of RLRs in GEN2.2 cells. (**A-H**) Cells were transfected with NLRC5, NLRX1 or scrambled (scr, negative control) siRNAs. After 24 hours cells were stimulated with 0.25 μM CpG-A for 16 hours and the efficacy of gene silencing (**A-D**) and the protein levels of RIG-I, MDA5 and MAVS (**A,C,E,G**) were assessed by western blot analysis. Representative blots are shown. Bar graphs represent silencing efficiency of NLRC5 (**B**) and NLRX1 (**D**), and the protein levels of RIG-I, MDA5 and MAVS (**F**,**H**) as compared with scrambled, where scrambled is taken as 100 percent. (**B**,**D,F,H**) The mean ± SD of 4-8 independent experiments are shown. Statistical analysis was performed by Student's t-test. ****p<0.0001


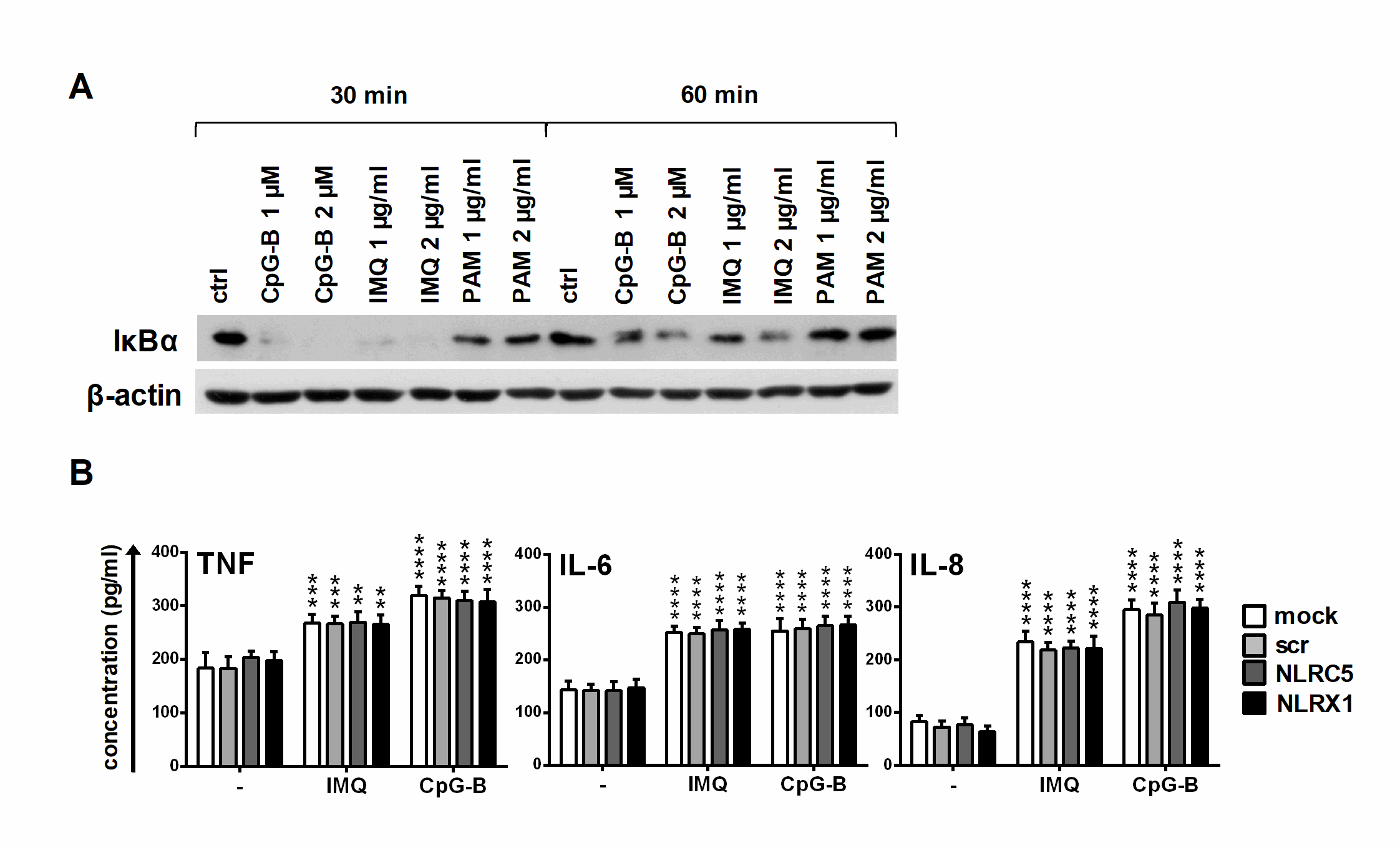


**Supplementary Figure 2.** NLRC5 and NLRX1 does not influence the activity of NF-κB signaling pathway in GEN2.2 cells exposed to different TLR agonists. (**A**) Cells were activated with TLR9 agonist (CpG-B), TLR7 agonist (Imiquimod; IMQ) and TLR 1/2 ligand (PAM3CSK4; PAM) at the indicated concentrations, and the kinetics of IκBα degradation was determined by western blotting. (**B**) Cells were transfected with siRNAs specific for NLRC5, NLRX1 or scrambled (scr) siRNAs for 24 hours then treated with different TLR agonist (IMQ, CpG-B) and after 24 hours the secreted TNF, IL-6 and IL-8 (**C**) levels were measured by ELISA. (**A**) A representative blot is shown. (**B**) Data are represented as means ± SD of three individual experiments and analyzed using one-way ANOVA followed by Bonferroni’s post-hoc test. **p<0.01, ***p<0.001 ****p<0.0001 vs untreated


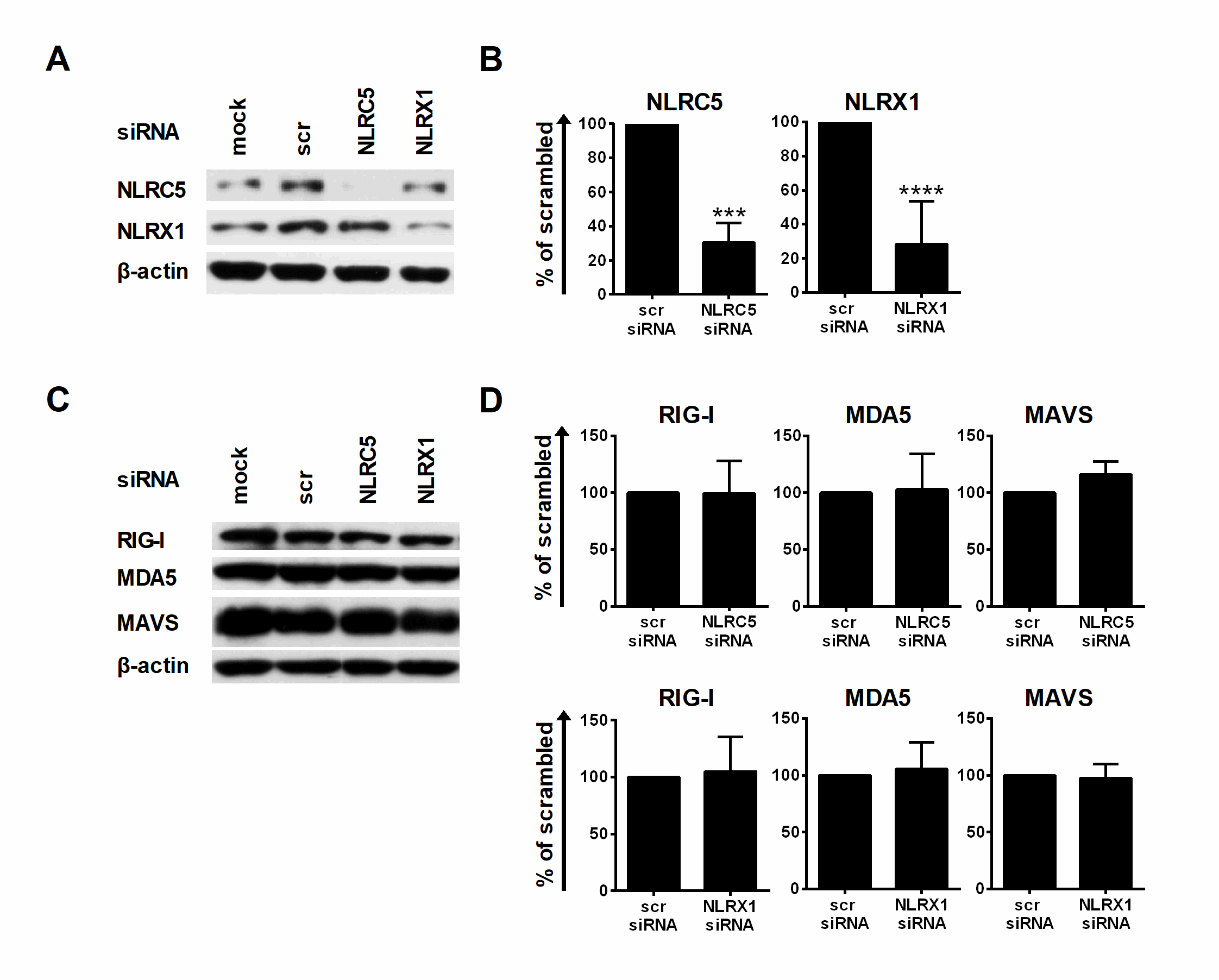


**Supplementary Figure 3.** Silencing of NLRC5 and NLRX1 does not affect the expression of RLRs in human immature moDCs. (**A-D**) Freshly isolated monocytes were transfected with the indicated siRNAs and differentiated into moDCs. On day 5, the efficacy of gene silencing (**A,B**) and the protein levels of RIG-I, MDA5 and MAVS (**C,D**) were assessed by western blot analysis. (**A,C**) Representative blots are shown. Bar graphs represent the silencing efficiency of NLRC5 and NLRX1 (**B**), and the protein levels of RIG-I, MDA5 and MAVS in silenced cells (**D**), where the values are expressed as the percentage of scrambled siRNA transfected cells. (**B,D**) Data are shown as means ± SD of 4-9 independent measurements and statistical analysis was performed by Student's t-test. ***p<0.001, ****p<0.0001


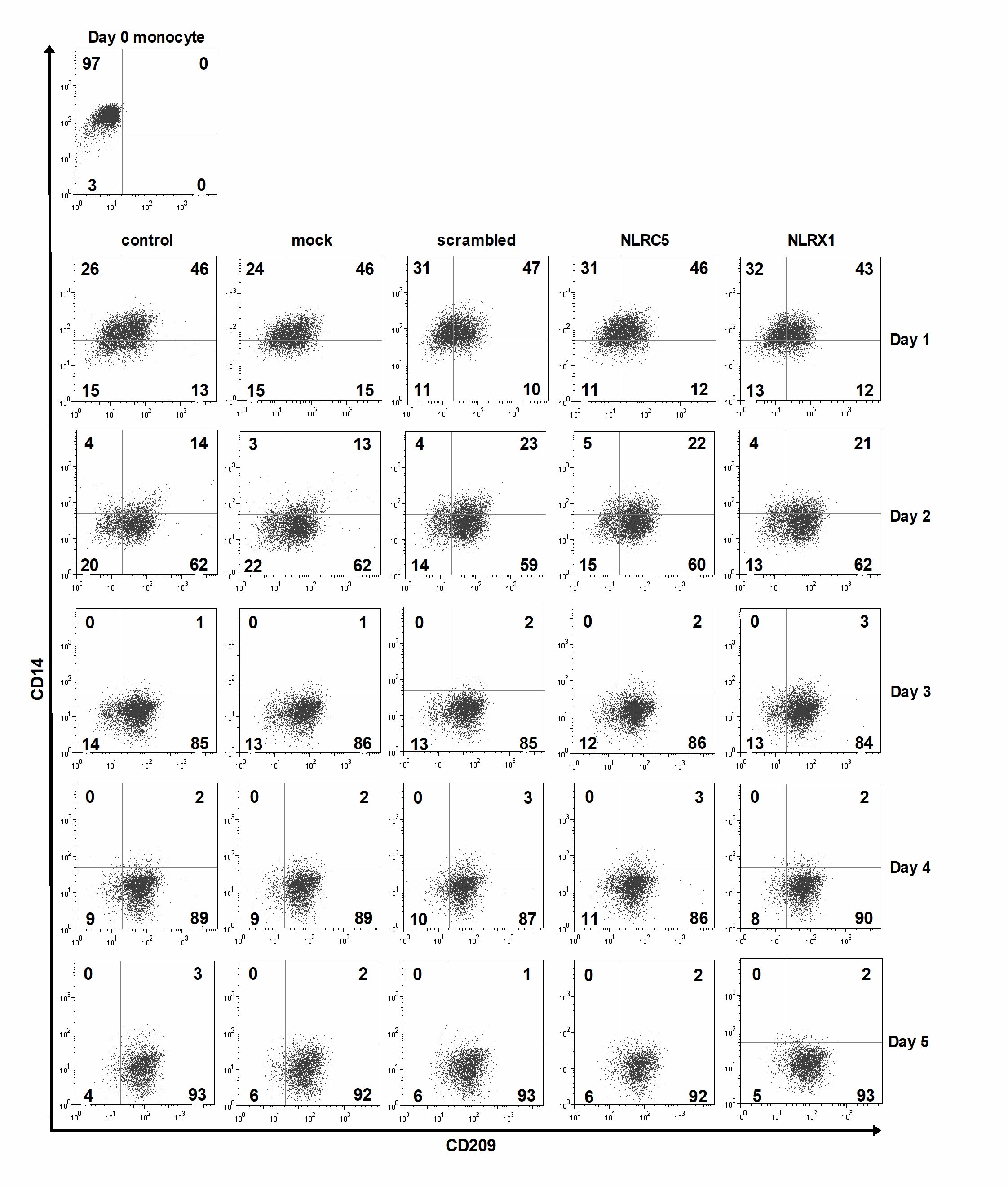


**Supplementary Figure 4.** Silencing of NLRC5 and NLRX1 does not affect the differentiation process of human moDCs. Freshly isolated monocytes were transfected with the indicated siRNAs at day 0 and differentiated into moDCs. The changes in the expression levels of CD14 and CD209 cell surface proteins were monitored during the differentiation (from day 0 to day 5) by flow cytometry. Representative dot blots are shown where gates were determined by using relevant isotype control antibodies. Numbers indicate the percentages of positive cells inside the quadrant gates.


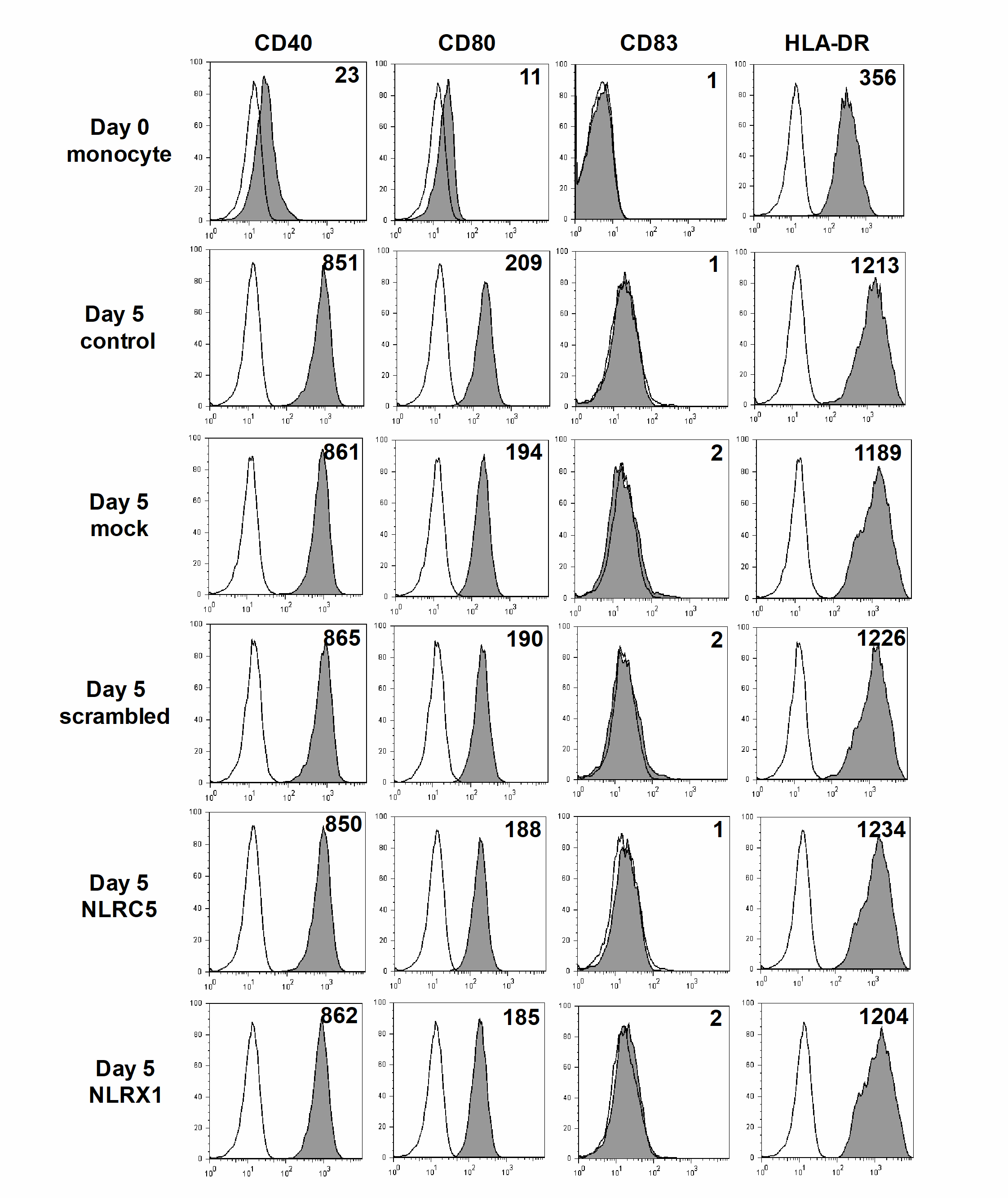


**Supplementary Figure 5.** 5-day moDCs silenced for NLRC5 or NLRX1 display normal immature DC phenotype. Freshly isolated monocytes were transfected with the indicated siRNAs at day 0 and differentiated into moDCs. On day 5, the expression levels of CD40, CD80, CD83 and HLA-DR were determined by using flow cytometric analysis. Representative histograms are shown from 3 individual experiments. Unfilled histograms correspond to isotype controls whereas filled histograms represent staining with specific antibody. Numbers indicate the delta median fluorescence intensities (MFI) of the samples calculated by subtracting the values of isotype control from the values of samples with specific antibody staining.
